# Recoding by *N*^*1*^-methylpseudouridine guides rational mRNA vaccine design

**DOI:** 10.1038/s41467-026-77796-3

**Published:** 2026-09-28

**Authors:** Panagiotis Poulis, Giovanni Robecchi, Arina O. Kurochkina, Michele Felletti, Marina V. Rodnina

**Affiliations:** Department of Physical Biochemistry, Max Planck Institute for Multidisciplinary Sciences, Göttingen, Germany

**Keywords:** Ribosome, RNA, Single-molecule biophysics

## Abstract

*N*^*1*^-methylpseudouridine (m^1^Ψ) is a key modification used in SARS-CoV-2 mRNA vaccines that reduces immunogenicity and increases mRNA stability. Recent studies suggested that m^1^Ψ can promote ribosomal frameshifting, a translational error generating aberrant peptides that elicit immune responses, raising concerns about unintended antigenicity. Here, we systematically examined the efficiency of frameshifting induced by m^1^Ψ and the underlying mechanism, aiming to inform future mRNA vaccine design. Using mRNA-based dual-fluorescence reporters in cells, fully in vitro reconstituted translation system, and single-molecule FRET microscopy, we show that m^1^Ψ increases +1 frameshifting on UUUC motifs in therapeutic mRNAs. Frameshifting occurs when the peptidyl-tRNA pauses in the ribosomal P site, where m^1^Ψ both weakens codon-anticodon interactions and promotes a frameshifting-prone tRNA conformation. Replacing slippery UUUC motifs with UUCC or UUUU eliminates this effect. Our results reveal how a clinically relevant mRNA modification promotes recoding and show that codon optimization mitigates this risk in therapeutic mRNA design.

## Abstract (web summary)

*N*^*1*^-methylpseudouridine (m^1^Ψ) is crucial for mRNA vaccine development but can trigger ribosome frameshifting. Here the authors show that m^1^Ψ destabilizes codon-anticodon pairing and P-site tRNA dynamics on slippery sequences, driving frameshifting, a risk that codon optimization can mitigate.

## Introduction

**Fig. 1:**
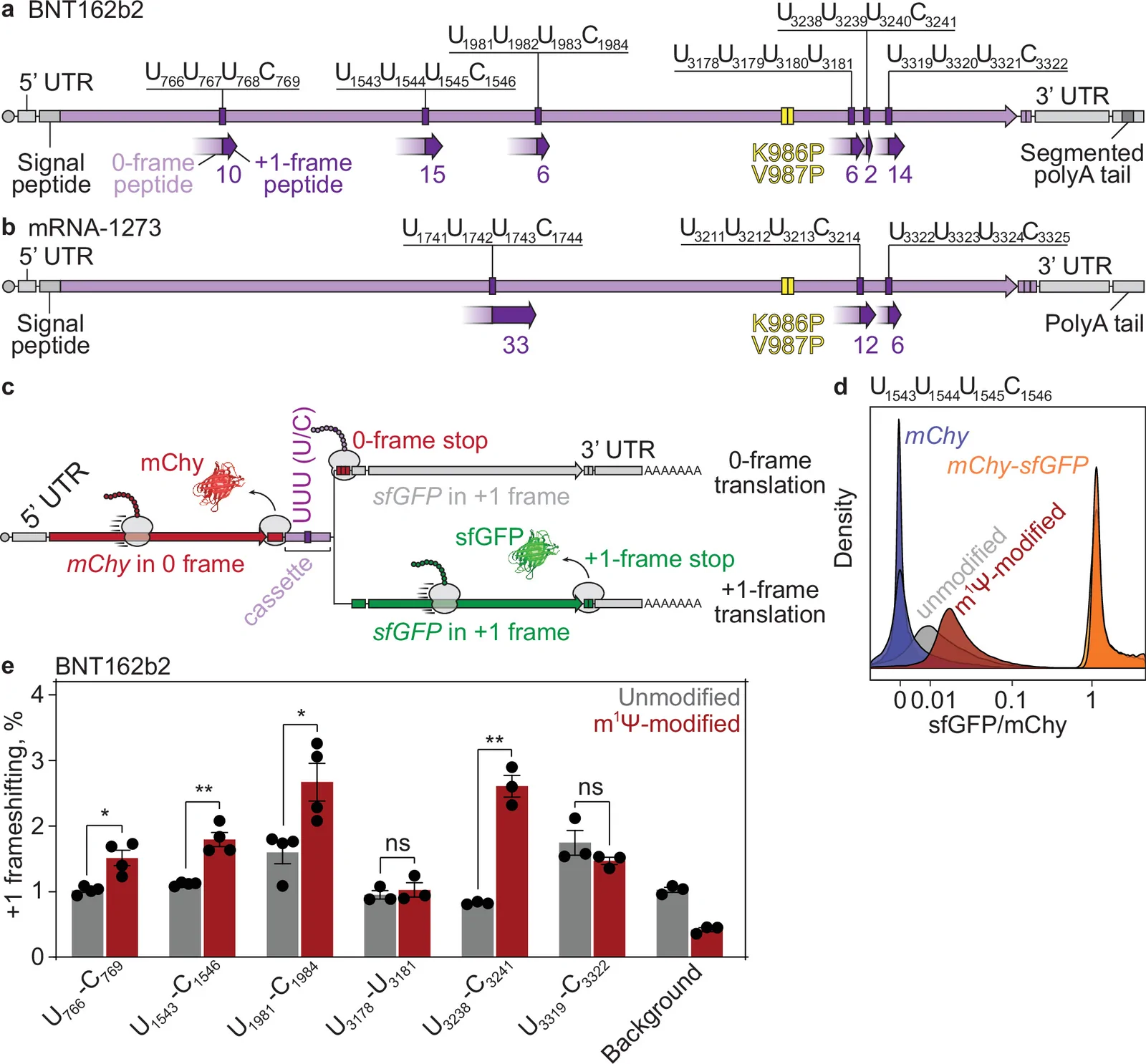
m^1^Ψ-induced +1 frameshifting on slippery sequences of vaccine reporter mRNAs in human cells. Schematics of BNT162b2 (**a**) and mRNA-1273 (**b**) vaccine mRNAs encoding a codon-optimized SARS-CoV-2 spike protein variant (lilac) containing a signal peptide (gray) and the K986P and V987P mutations (yellow) followed by stop codons (lilac squares). The putative +1 frameshifting-prone slippery sequences (purple squares; nucleotides above indicate slippery codons) may, upon +1 frameshifting, produce +1-frame peptides (purple arrows). Numbers in purple indicate the length in amino acid residues (aa) of +1-frame peptide extensions up to the first +1-frame stop codon. **c** Schematic of the dual-fluorescence mRNA reporter to monitor +1 frameshifting in vivo. In the absence of +1 frameshifting on the slippery sequence (purple) of the cassette (lilac), translation of *mChy* (red) terminates at 0-frame stop codons (red boxes; upper branch). Upon +1 frameshifting, the 0-frame stop codons are omitted, and translation resumes in the +1 frame to produce sfGFP (green; lower branch). Part of the figure was created in BioRender. https://BioRender.com/cyswms8. **d** Representative sfGFP/mChy ratio distribution after transfection of HEK293T with mRNA reporters containing only *mChy* (sfGFP/mChy = 0 indicates 0% frameshifting; light blue for unmodified; dark blue for m^1^Ψ-modified), *sfGFP* in-frame to *mChy* (*mChy-sfGFP*; sfGFP/mChy = 1 indicates 100% frameshifting, light orange for unmodified and dark orange for m^1^Ψ-modified), and cassette carrying the U_1543_U_1544_U_1545_C_1546_ slippery sequence between *mChy* and +1-frame *sfGFP* (gray for unmodified; maroon for m^1^Ψ-modified). **e** +1 frameshifting on the slippery motifs of BNT162b2 after transfection with unmodified (gray) and m^1^Ψ-modified (maroon) mRNA reporters. Shown are values in individual replicates (black circles) and mean values with error bars representing standard error (SE) from *n* = 3 (U_3178_-U_3181_, U_3238_-C_3241_, U_3319_-C_3322_, background) and *n* = 4 (U_766_-C_769_, U_1543_-C_1546_, U_1981_-C_1984_) independent biological replicates. Statistical significance by two-tailed Welch’s *t*-test: *P = 0.0220 (*t* = 4.005; degrees of freedom (df) = 3.405); **P = 0.0072 (*t* = 6.324; df = 3.109); *P = 0.0247 (*t* = 3.209; df = 4.859); ns not significant, *P* = 0.5890 (*t* = 0.5980; df = 3.248); **P = 0.0084 (*t* = 10.68; df = 2.019); ns, *P* = 0.2782 (*t* = 1.402; df = 2.358). Exact values are summarized in Supplementary Table 1. Source data are provided as a Source Data file.

The introduction of *N*^*1*^-methylpseudouridine (m^1^Ψ) into mRNA marked a breakthrough in the development of RNA vaccines, enabling potent immunization strategies against COVID-19^1^. A key property of m^1^Ψ is the dampening of signaling cascades triggered by exogenous unmodified RNA in human cells, which result in cytokine production and translation shutdown^1–3^, thereby increasing the stability of the modified mRNA^2–5^. Vaccine mRNAs are produced by in vitro transcription in the presence of m^1^Ψ triphosphate from DNA templates containing a T7 transcription promoter, 5′ and 3′ untranslated regions (UTRs), and an open reading frame (ORF) encoding a prefusion-stabilized variant of the SARS-CoV-2 spike protein (Fig. 1a)^2,6^. The mRNAs are co-transcriptionally 5′-capped, 3′-polyadenylated, and encapsulated in lipid nanoparticles^7^, which deliver them into the cytoplasm of human cells^8^. There, m^1^Ψ-modified mRNA is translated into SARS-CoV-2 spike proteins, which are processed by the proteasome for presentation on major histocompatibility complexes (MHC) to activate cytotoxic T cells, or are secreted to activate B cells or helper T cells^6,8–11^. This immune response provides strong, rapid, and multi-target protection against SARS-CoV-2 infection^9–15^. The success and versatility of this approach have since spurred global efforts to extend mRNA vaccine technology to other infectious diseases and cancer^16–21^.

Naturally occurring mRNA modifications are dynamically installed by “writers”, removed by “erasers”, and recognized by dedicated “readers”, including the ribosome. These epitranscriptomic marks regulate diverse aspects of gene expression. Numerous studies have shown that such modifications can affect both translation efficiency^22^ and fidelity^23–26^. In contrast, m^1^Ψ, which does not occur naturally in human mRNA, is incorporated synthetically into therapeutic mRNAs, and the ribosome appears to be its only reader. Although initial studies reported no effect of m^1^Ψ on translation errors^27^, recent studies linked m^1^Ψ to translational miscoding that produces amino acid substitutions in proteins^24^, as well as to +1 ribosome frameshifting^28^ – a recoding event in which the ribosome shifts by one nucleotide toward the 3′ end of the mRNA into the +1 frame^29^. Frameshifting typically occurs on slippery tetrameric XXX (X/Y) motifs, where the same tRNA can base-pair with both the 0-frame and +1- or –1-frame codons. Translation in the trans-frame produces peptides with altered amino acid sequences that may elicit unintended immune responses following vaccination with m^1^Ψ-modified mRNA. Indeed, +1-frame peptides have been identified by mass spectrometry after translation of the SARS-CoV-2 Delta (B.1.617.2) spike mRNA^30^, and T cells primed against +1-frame peptides derived from BNT162b2 mRNA were detected in vaccinated individuals^28^. Despite the importance of m^1^Ψ in therapeutic mRNAs, its effect on translation errors, the mechanism by which it promotes +1 frameshifting, the role of the slippery sequence, and strategies to mitigate such recoding events in human cells remain poorly understood.

## Results

### m^1^Ψ induces low-level+1 frameshifting

The BNT162b2 mRNA (Pfizer-BioNTech) contains six slippery motifs that can support +1 frameshifting (U_766_U_767_U_768_C_769_, encoding Phe238; U_1543_U_1544_U_1545_C_1546_, Phe497; U_1981_U_1982_U_1983_C_1984_, Phe643; U_3178_U_3179_U_3180_U_3181_, Phe1042; U_3238_U_3239_U_3240_C_3241_, Phe1062; U_3319_U_3320_U_3321_C_3322_, Phe1089; Fig. 1a), while mRNA-1273 (Moderna) contains three such motifs (U_1741_U_1742_U_1743_C_1744_, Phe562; U_3211_U_3212_U_3213_C_3214_, Phe1052; U_3322_U_3323_U_3324_C_3325_, Phe1089; Fig. 1b) within the open reading frame. To test the +1-frameshifting efficiency of these sites in vivo, we employed a dual-fluorescence reporter assay in human embryonic kidney 293T (HEK293T) cells. Reporter mRNAs contained the 5′ and 3′ UTRs of BNT162b2 or mRNA-1273 mRNA, followed by an ORF encoding mCherry (mChy) in the 0 frame and a downstream ORF encoding superfolder Green Fluorescent Protein (sfGFP) in +1 frame (Fig. 1c). The two ORFs are separated by a cassette containing one of the slippery UUU (U/C) motifs of BNT162b2 or mRNA-1273, flanked by 10 codons upstream and codons up to the first +1-frame stop codon downstream (Fig. 1a–c). The cassette was positioned in-frame with *mChy* and embedded between P2A (in 0 frame) and T2A (in +1 frame) StopGo elements, ensuring undisturbed maturation and fluorescence of both reporters (Fig. 1c). Translation of the test cassette in 0 frame terminated at three consecutive stop codons, whereas +1 frameshifting allowed translation to bypass these stops and produce +1-frame sfGFP (Fig. 1c). As controls, we prepared an mRNA lacking the test cassette (Supplementary Table 1), which was used to define the background signal and lower detection limit of the assay, and mRNAs containing only 0-frame mChy or sfGFP in-frame with mChy, corresponding to 0% and 100% +1 frameshifting, respectively (Supplementary Fig. 1a). Cells were transfected with either unmodified or m^1^Ψ-modified mRNAs, allowing direct comparison of frameshifting events.

We quantified +1 frameshifting as the median of the sfGFP/mChy fluorescence ratio, normalized to the ratio median of the sfGFP/mChy = 1 control mRNA (“Methods” section, Fig. 1d and Supplementary Fig. 1b–d). Frameshifting efficiency on BNT162b2 sequences showed a strong dependence on the fourth nucleotide of the slippery sequence (U or C), ranging from 0.82 ± 0.02% to 1.7 ± 0.3% for the unmodified mRNA reporters and from 1.0 ± 0.2% to 2.7 ± 0.5% for m^1^Ψ-modified reporters (Fig. 1d, e and Supplementary Table 1). Removing potential sites of internal initiation, such as AUG of *sfGFP* and near-cognate start codon CUG in T2A, did not affect +1 frameshifting, indicating that sfGFP production results from +1 frameshifting rather than unintended internal initiation (Supplementary Fig. 1e). These results confirm that low-level +1 frameshifting occurs during translation of m^1^Ψ-modified mRNAs, consistent with previous observations of +1-frame products detected by mass spectrometry^30^, increased frameshifting in reporter assays, and the presence of +1-frame-specific cytotoxic T cells in vaccinated individuals^28^. Notably, four out of the six slippery UUU (U/C) motifs in BNT162b2 showed increased +1 frameshifting, while two did not (Fig. 1e), indicating that the slippery sequence alone is not sufficient to determine frameshifting efficiency. We obtained similar results on the slippery sequences of mRNA-1273 (Moderna; Supplementary Fig. 2a, b), although accurate calculation of +1 frameshifting was not possible due to low translation efficiency. Notably, the +1 frameshifting efficiencies in our experiments are 3- to 7-fold lower than in previous studies^28^, highlighting differences in sensitivity of the various reporter assays to measure recoding events, but agree with recent studies reporting frameshifting efficiency <5%, the detection limit for frameshifting using ribosome profiling^31^.

Using the same approach, we tested whether m^1^Ψ can also promote stop codon readthrough by near-cognate aa-tRNA on the original BNT162b2 sequence bearing two consecutive UGA stop codons, leading to continued translation into the 3′ UTR (Supplementary Fig. 3a), as suggested for pseudouridine^25,26^. Because the readthrough efficiency in unmodified and m^1^Ψ-modified mRNA reporters is close to the detection limit (Supplementary Fig. 3b, c and Supplementary Table 1), we conclude that m^1^Ψ does not promote readthrough above the ≈1% detection limit of our assay during translation of BNT162b2 vaccine mRNA.

### m^1^Ψ does not affect mRNA decoding

We next explored the molecular mechanism of +1 frameshifting. In principle, slippage could occur either during decoding of the slippery codon, as suggested by Mulroney et al.^28^, or at post-decoding steps, including ribosome translocation to the next codon^32–36^ or during the waiting periods before and after translocation, when the ribosome is particularly prone to slippage^37,38^. As the effect of m^1^Ψ on decoding has not been examined, we first monitored decoding of UUU C or m^1^Ψm^1^Ψm^1^Ψ C slippery codons by its cognate Phe-tRNA^Phe^ in real time by smFRET. Because the core mechanism of decoding is highly conserved from bacteria to mammals, for these experiments, we used *E. coli* ribosomes labeled with Cy3 at the ribosomal protein L11 and Cy5 on Phe-tRNA^Phe^ (Supplementary Fig. 4a) in a total internal reflection fluorescence (TIRF) microscopy setup^22^. Ribosome complexes carrying fMet-Ala-tRNA^Ala^ in the P site and exposing UUU C or m^1^Ψm^1^Ψm^1^Ψ C codon in the vacant A site (POST, FRET 0.0) were immobilized on coverslips via a 5′-biotinylated mRNA (Supplementary Fig. 4a). Upon addition of EF-Tu–GTP–Phe-tRNA^Phe^ ternary complex (TC), FRET trajectories reported initial binding (IB), codon reading (CR), and Phe-tRNA^Phe^ accommodation in the A site, together with subsequent post-decoding fluctuations of peptidyl-tRNA between A/A, A/P, and A/P* states on single ribosomes^22,39,40^.

Among the trajectories, we observed four classes of traces. The first class included trajectories reaching a short-lived high FRET state followed by a rapid return to FRET 0.0 (Supplementary Fig. 4b). Population analysis revealed a single state with FRET ∼0.8 corresponding to initial binding of the ternary complex followed by rapid tRNA rejection without progression to accommodation (Supplementary Fig. 4c, d). The second class reported complete trajectories of decoding, beginning with a short-lived high FRET state that rapidly transitioned to interconversions between two lower FRET states (Supplementary Fig. 4e). Population distribution analysis of these trajectories showed three states: CR, followed by rapid accommodation to the A/A state and subsequent fluctuations between A/A, A/P (FRET ∼0.8), and A/P* (FRET ∼0.6) states (Supplementary Fig. 4f, g). The third class of traces displayed only these latter fluctuations (Supplementary Fig. 4h), corresponding to ribosome complexes that had completed decoding before imaging (Supplementary Fig. 4i, j). Finally, a small fraction of trajectories showed a long-lived FRET state representing ribosomes stalled at the CR state without progression to accommodation. The relative abundance of these four classes, which is a proxy for the decoding efficiency, did not differ significantly between UUU C and m^1^Ψm^1^Ψm^1^Ψ C codons (Supplementary Fig. 5a and Supplementary Table 2).

We also examined whether decoding rates were altered. From the traces showing rapid rejection, we extracted the dwell time of IB (*τ*_*−1*_), while from decoding traces we calculated the CR dwell time before accommodation (*τ*_*CR→forward*_). We also quantified interconversions between A/A-A/P and A/P* states (*τ*_*0.8→0.6*_ and *τ*_*0.6→0.8*_). None of these dwell times differed significantly between modified and unmodified codons (Supplementary Fig. 5b and Supplementary Table 2), indicating that m^1^Ψ does not affect decoding dynamics or post-decoding peptidyl-tRNA^Phe^ fluctuations, in agreement with previous studies^24^.

Next, we tested whether m^1^Ψ affects codon–anticodon stability during decoding, as reported for other mRNA modifications^22^. We repeated smFRET experiments using the GTPase-deficient EF-Tu(H84A) mutant, which blocks progression beyond GTPase activation, but does not affect any of the preceding steps^41^, making dissociation of tRNA from the CR state rate-limiting for the return to FRET 0.0 state^22,41^ (Supplementary Fig. 5c). As expected, most (∼80%) ribosomes stalled at a long-lived high FRET state before returning to baseline (Supplementary Fig. 5d, e and Supplementary Table 2). Population analysis confirmed a single CR state (FRET ∼1) (Supplementary Fig. 5f, g). The dwell times of the CR state (*τ*_*−2*_) were indistinguishable between UUU C and m^1^Ψm^1^Ψm^1^Ψ C codons (Supplementary Fig. 5h), showing that m^1^Ψ does not affect codon–anticodon stability in the A site.

Finally, to test whether availability of the tRNA decoding the slippery codon influences frameshifting, we performed codon walk experiments^32^ using mRNAs with slippery UUU C or m^1^Ψm^1^Ψm^1^Ψ C sequences (Supplementary Fig. 6a). Translation in 0 frame produces the fMet-Ala-Phe-Arg (fMAFR) peptide, while +1 frameshifting generates fMet-Ala-Phe-Val (fMAFV; Supplementary Fig. 6a), which are separated via HPLC and quantified by [^3^H]fMet and [^14^C]Arg scintillation counting (“Methods” section and Supplementary Fig. 6b–d). Reducing Phe-tRNA^Phe^ concentration decreased 0-frame translation, as expected, but did not increase +1-frame products (Supplementary Fig. 6e, f), indicating that ribosome stalling from slow decoding of m^1^Ψ-modified codons is unlikely to drive frameshifting. Together, these data strongly suggest that m^1^Ψ-induced frameshifting does not occur during the decoding of slippery codons.

### P-site stalling drives frameshifting by m^1^Ψ

**Fig. 2:**
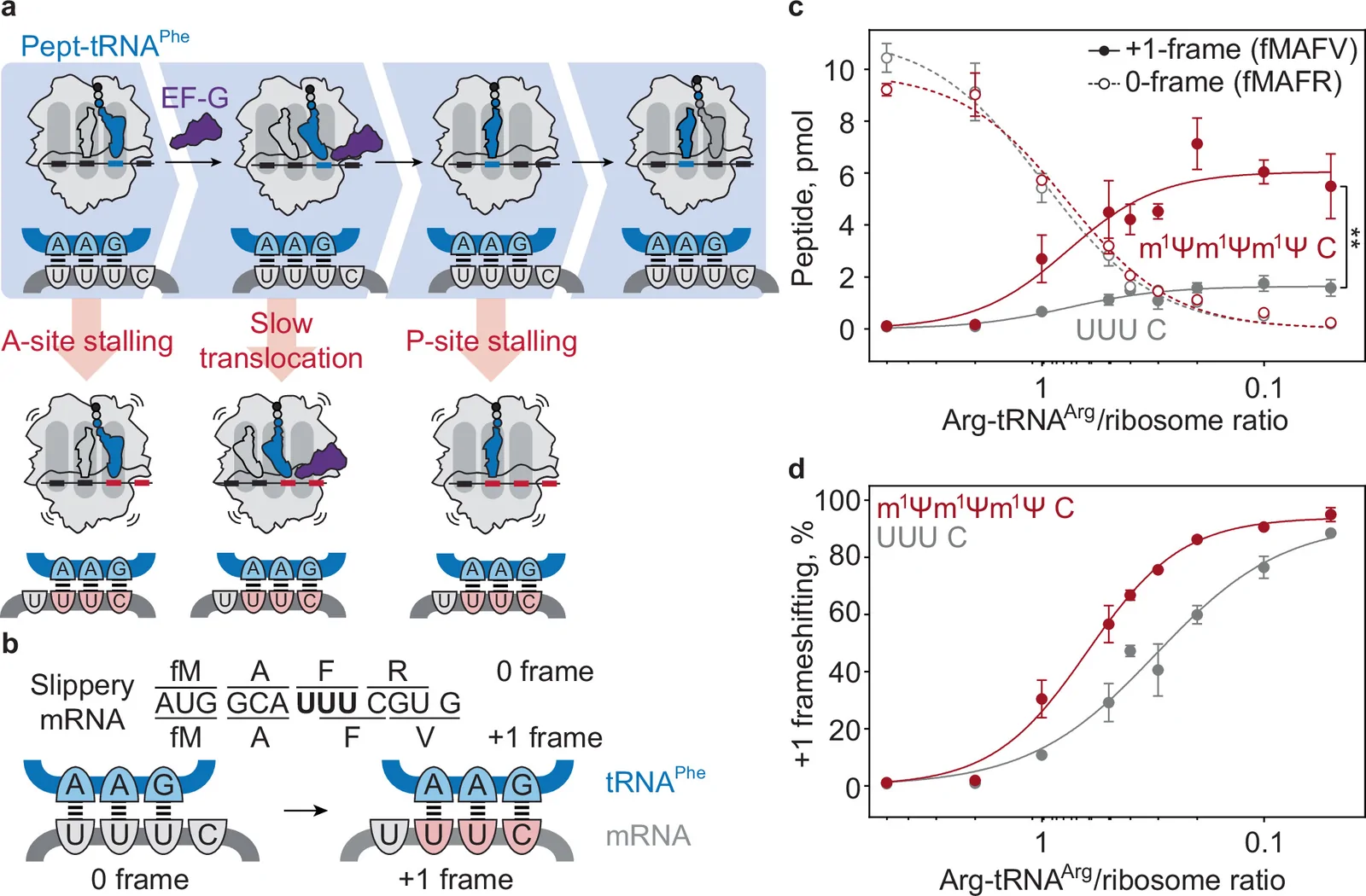
Mechanism of +1 frameshifting on m^1^Ψ-modified slippery codons. **a** Schematic overview of potential mechanisms that can drive ribosome frameshifting. Frameshifting on slippery codons (gray) can be induced by either tRNA stalling at the A site, impaired translocation, or tRNA stalling at the P site due to limited availability of the aminoacyl-tRNA cognate to the downstream 0-frame codon. +1-frame codon, red; EF-G, purple; tRNA^Phe^, blue. Codons (solid bold lines) are shown in respective colors with the tRNAs and the amino acids (circles). Blue shading indicates 0-frame translation; red arrows indicate the frameshifting pathway. **b** Coding sequence of mRNA carrying the slippery UUU C (or m^1^Ψm^1^Ψm^1^Ψ C, bold) sequence that allows base-pairing with tRNA^Phe^ anticodon (blue) in +1 frame (red). **c** m^1^Ψ stimulates “hungry” frameshifting. Synthesis of 0-frame fMAFR (open circles and dotted lines) and +1-frame fMAFV (closed circles and solid lines) products during translation of UUU C (gray) and m^1^Ψm^1^Ψm^1^Ψ C (maroon) sequences at decreasing Arg-tRNA^Arg^ to ribosome ratio (note the reverse X-axis for the Arg-tRNA^Arg^/ribosome ratio). Shown are mean values with error bars representing SE of *n* = 3 independent biological replicates. Asterisks represent the statistically significant differences in +1-frame peptide synthesis according to a two-way repeated-measures ANOVA test. **P = 0.0028 (F(1, 4) = 42.99; df = 1). Lines are hyperbolic functions (“Methods” section). **d** +1 frameshifting efficiency (%) calculated from the data in (**c**). Shown are mean values with error bars representing SE from *n* = 3 independent biological replicates. Lines are hyperbolic functions (“Methods” section). Source data are provided as a Source Data file.

Efficient +1 frameshifting could, in principle, arise at several post-decoding steps^29^ (Fig. 2a). It may occur, for instance, during prolonged residence of the peptidyl-tRNA in the A site prior to translocation^34,42,43^. In this scenario, a longer A-site dwell time of the peptidyl-tRNA is expected to increase the probability of frameshifting. To mimic such A-site stalling, we used reduced concentrations of EF-G, thereby increasing the waiting time before translocation. Frameshifting efficiency was then quantified from the ratio of 0- and +1-frame peptides synthesized in the reaction. In a positive control, –1 frameshifting-prone A AAA AAG sequence showed the expected increase in –1 frameshifting under EF-G depletion^34^ (Supplementary Fig. 7a). By contrast, m^1^Ψm^1^Ψm^1^Ψ C did not show increased +1 frameshifting while waiting for EF-G to arrive (Supplementary Fig. 7b, c), arguing against an A-site peptidyl-tRNA stalling mechanism for frameshifting.

Frameshifting could also occur during tRNA-mRNA translocation. During this process, tRNAs move from the A and P sites to the P and E sites of the ribosome via a series of intermediates. One of these, the chimeric (CHI; ap/P and pe/E) state, is particularly prone to frameshifting. In the CHI state, the anticodon of peptidyl-tRNA resides between the A and P sites of the small ribosomal subunit^44,45^, resulting in loss of key stabilizing contacts with the ribosome. During rapid translocation, the CHI state is short-lived, providing insufficient time for the tRNA anticodon to explore alternative frames. However, when exit from the CHI state is delayed, the peptidyl-tRNA anticodon may slip into an alternative frame^44–46^. In this scenario, translocation-defective EF-G mutants are expected to enhance the effect of m^1^Ψ on frameshifting, as observed in viral –1 frameshifting on unmodified mRNAs^32,46^. To increase frameshifting during translocation, we used the EF-G(Q507D) mutant, which strongly promotes –1 frameshifting on A AAA AAG by delaying exit from the CHI state^34,35^ (Supplementary Fig. 7d). However, +1 frameshifting on m^1^Ψm^1^Ψm^1^Ψ C remained low (Supplementary Fig. 7e), indicating that impaired translocation does not contribute to the observed frameshifting.

Finally, frameshifting may also occur during P-site stalling of the peptidyl-tRNA, when delivery of the next aa-tRNA is delayed – for instance, due to low abundance of the cognate tRNA or an inaccessible A-site decoding center. Under such conditions, the P-site peptidyl-tRNA has more time to sample alternative, out-of-frame codons^37,38^. To test the contribution of this “hungry” frameshifting mechanism, we limited the availability of Arg-tRNA^Arg^, the cognate tRNA for the downstream 0-frame CGU codon when peptidyl-tRNA^Phe^ is bound to the slippery codon in the P site (Fig. 2b). With decreasing Arg-tRNA^Arg^ availability, +1 frameshifting on m^1^Ψm^1^Ψm^1^Ψ C increased sharply, from ∼1% at high Arg-tRNA^Arg^ concentrations to >90% at sub-stoichiometric conditions (Fig. 2c). UUU C required more severe tRNA depletion to reach comparable frameshifting efficiencies, showing that m^1^Ψm^1^Ψm^1^Ψ C drives frameshifting more readily when tRNA supply is limiting (Fig. 2d). Importantly, modulation of +1-frame Val-tRNA^Val^ did not affect +1-frame translation (Supplementary Fig. 7f), confirming that the effect depends on the availability of the 0-frame Arg-tRNA^Arg^. We also tested whether elongation factor P (EF-P), an accessory factor^47,48^ known to stabilize the reading frame on +1 frameshifting-prone CCC C sequences^49^, can regulate frameshifting under limiting Arg-tRNA^Arg^ conditions (Supplementary Fig. 8a). However, EF-P did not prevent +1 frameshifting on m^1^Ψm^1^Ψm^1^Ψ C (Supplementary Fig. 8b, c).

Previous studies have implicated “hungry” P-site stalling as a mechanism for –1 frameshifting^37,38^, prompting us to test whether stalling of tRNA^Phe^ on the –1 frameshifting-prone U UUC (or m^1^Ψ m^1^Ψm^1^ΨC) codons – present four times in BNT162b2 and once in mRNA-1273 (Supplementary Fig. 9a, b) – could promote –1 frameshifting. To examine this, we translated a slippery mRNA encoding 0-frame fMAFV and –1-frame fMAFR (Supplementary Fig. 9c) under decreasing Val-tRNA^Val^ concentration. However, reduced availability of the cognate A-site tRNA did not enhance –1-frame translation; instead, m^1^Ψ may even be slightly protective against –1 frameshifting (Supplementary Fig. 9d, e). Consistent with this, –1 frameshifting on the U UUC sequences in BNT162b2 was only marginally increased in m^1^Ψ-modified reporter mRNAs in human cells using the dual-fluorescence reporter assay (Supplementary Fig. 9f, g). The low –1 frameshifting efficiency on U UUC sequences is likely due to the thermodynamic preference of tRNA^Phe^ for UUC, rather than UUU^50^, whereas canonical frameshifting sequences – such as U UUU or heptameric U UUU UU(U/C) motifs – are absent in BNT162b2 and mRNA-1273.

In summary, our results demonstrate that +1 ribosome frameshifting on m^1^Ψ-modified slippery codons primarily occurs when the peptidyl-tRNA resides in the P site while awaiting delivery of the cognate tRNA for the next codon, representing a “hungry frameshifting” pathway.

### m^1^Ψ disrupts P-site tRNA dynamics

**Fig. 3:**
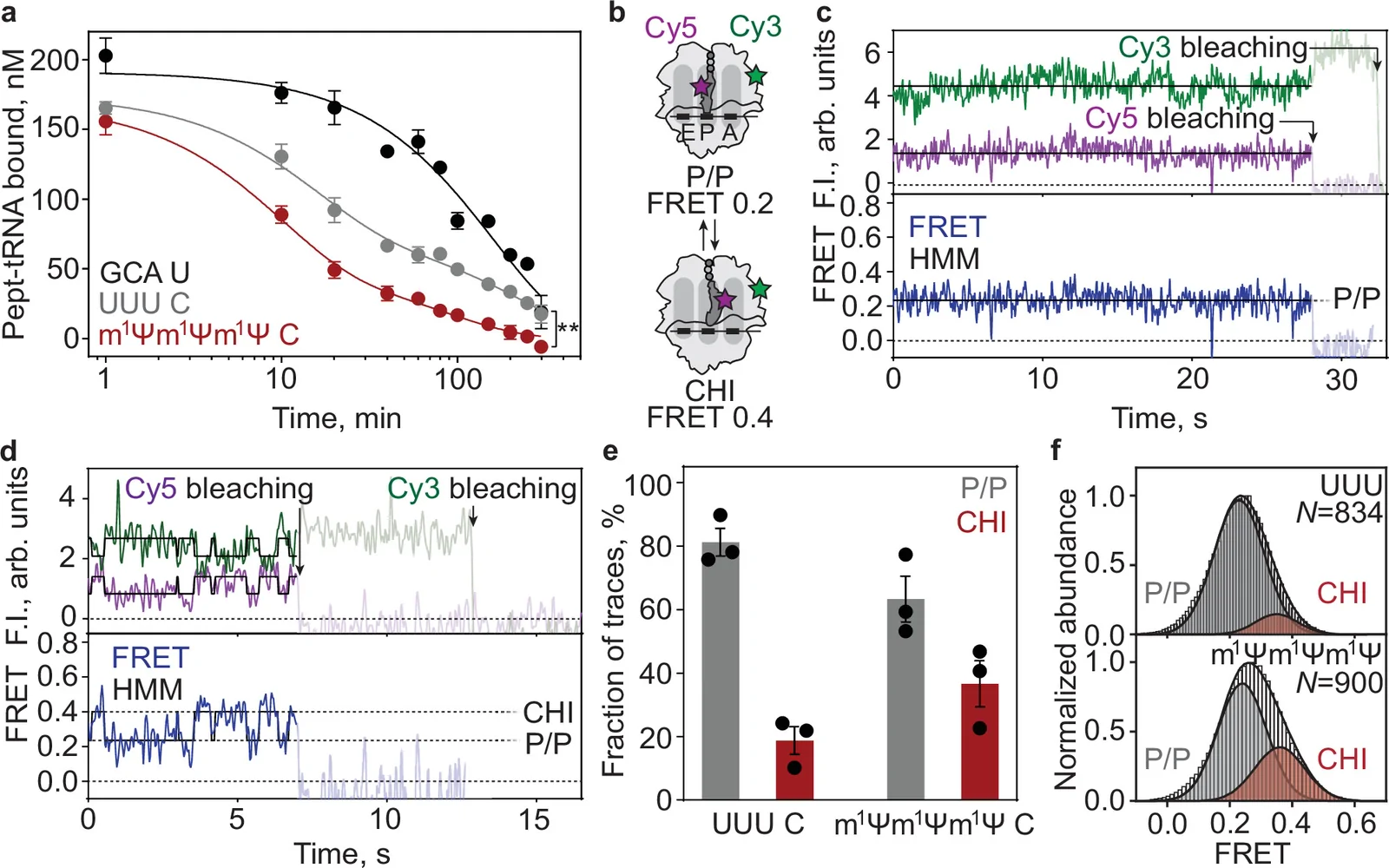
Codon–anticodon stability and non-canonical state of the P-site peptidyl-tRNA. **a** Codon–anticodon stability. Drop-off of P-site peptidyl-tRNA^Phe^ from the UUU C (gray) or m^1^Ψm^1^Ψm^1^Ψ C (maroon) codon and of P-site peptidyl-tRNA^Ala^ from the GCA U codon (black). Shown are mean values with error bars representing SE from *n* = 3 independent biological replicates. Asterisks represent statistically significant differences between UUU C and m^1^Ψm^1^Ψm^1^Ψ C, according to a two-way repeated-measures ANOVA test. **P = 0.0041 (F(1, 4) = 34.90; df = 1). Lines are single (black) or double (gray and maroon) exponential decay functions (“Methods” section). Exact values are summarized in Supplementary Table 3. **b** Schematic of smFRET experiment to monitor tRNA state in the P site. In the P/P state, peptidyl-tRNA^Phe^(Cy5) (purple star) resides far from L11-Cy3 (green star; FRET 0.2). Transition to the CHI state leads to an increase in FRET to 0.4. mRNA is shown as a solid line, with codons depicted as bold lines. **c** Static traces. Representative smFRET time trace of donor Cy3 (green) and acceptor Cy5 (purple) fluorescence intensities (F.I.), calculated FRET (blue), and hidden Markov modeling fit (HMM, black), corresponding to peptidyl-tRNA^Phe^ in the P/P state (FRET 0.2). arb. units, arbitrary units. **d** Dynamic traces. Same as (**c**) for traces showing reversible transitions between FRET 0.2 (P/P) and FRET 0.35 states (CHI). **e** Percentage of traces showing stable FRET 0.2 (P/P; gray) and fluctuations between FRET 0.2 and FRET 0.35 (CHI) states (maroon) on UUU C and m^1^Ψm^1^Ψm^1^Ψ C codons. Shown are values of individual replicates (black circles) and the mean with error bars representing SE from *n* = 3 independent biological replicates, each with *n* = 10 technical replicates. Exact values are summarized in Supplementary Table 3. Number of traces (*N*): P/P, *N* = 671 for UUU C, *N* = 576 for m^1^Ψm^1^Ψm^1^Ψ C; CHI, *N* = 163 for UUU C, *N* = 324 for m^1^Ψm^1^Ψm^1^Ψ C. **f** FRET population distribution histogram of the POST complex on UUU and m^1^Ψm^1^Ψm^1^Ψ, showing the relative abundance of the P/P (gray shade) and CHI (maroon shade) states. Shown are data from *n* = 3 biological replicates, with *n* = 10 technical replicates each. Exact values are summarized in Supplementary Table 3. Source data are provided as a Source Data file.

Having established that +1 frameshifting on m^1^Ψ-modified slippery codons occurs during P-site stalling, we next asked how the modification promotes slippage. We hypothesized that m^1^Ψ destabilizes the codon–anticodon interactions in the P site, making the duplex more prone to dissociation and re-pairing in the alternative frame. To test the stability of tRNA binding, we monitored dissociation of fMet-Ala-[^14^C]Phe-tRNA^Phe^ from the P site. Dissociation followed biphasic kinetics, with both fast and slow rates increased (1.5- and 2.75-fold, respectively) on m^1^Ψm^1^Ψm^1^Ψ C, compared to UUU C (Fig. 3a, Supplementary Fig. 10a and Supplementary Table 3), consistent with destabilization of the P-site codon–anticodon duplex in the presence of m^1^Ψ.

The two kinetic phases likely reflect distinct ribosome populations with different codon–anticodon duplex stabilities, indicating heterogeneity of the conformational states of the P-site tRNA on a slippery codon. Indeed, in contrast to UUU C and m^1^Ψm^1^Ψm^1^Ψ C, dissociation of P-site fMet-[^14^C]Ala-tRNA^Ala^ from the non-slippery GCA U codon follows single-phase kinetics, with a *k*_*off*_ similar to *k*_*slow*_^UUUC^ (Fig. 3a, Supplementary Fig. 10a and Supplementary Table 3), consistent with a homogeneous population with stable codon–anticodon duplexes. To identify the distinct ribosome populations, we performed smFRET experiments with L11(Cy3)-labeled ribosomes with fMet-Ala-Phe-tRNA^Phe^(Cy5) (POST; Fig. 3b). Among the trajectories, we again observed two classes of traces. One showed a single stable low FRET state (∼0.2) without transitions to higher or lower FRET (Fig. 3c and Supplementary Fig. 10b), corresponding to the authentic P/P state^51,52^ and consistent with slower tRNA dissociation rates (Fig. 3a). The other class of traces showed reversible transitions between low and higher FRET (∼0.35; Fig. 3d, Supplementary Fig. 10c and Supplementary Table 3). FRET 0.35 state has been previously characterized as the frameshifting-prone CHI state^35,51^, although we cannot exclude that it represents a structurally distinct non-canonical P state with the same FRET distance, akin to that found for tRNA^Pro^ on a +1 slippery sequence^53^. This dynamic ribosome population explains the fast kinetic phase in the tRNA dissociation experiments (Fig. 3a and Supplementary Fig. 10a). On the UUU C codon, 19 ± 6% of FRET traces showed P/P ↔ CHI fluctuations (Fig. 3e and Supplementary Table 3; 15 ± 6% in FRET population distribution analysis; Fig. 3f & Supplementary Table 3), whereas on m^1^Ψm^1^Ψm^1^Ψ C, these increase to 37 ± 10% (30 ± 8% in FRET population analysis; Fig. 3e, f, Supplementary Fig. 10d, e and Supplementary Table 3). Thus, m^1^Ψ favors peptidyl-tRNA fluctuations from the stable P/P state toward a non-canonical state, weakening the 0-frame pairing and favoring +1-frame re-pairing.

### Frameshifting modulation by codon context

**Fig. 4:**
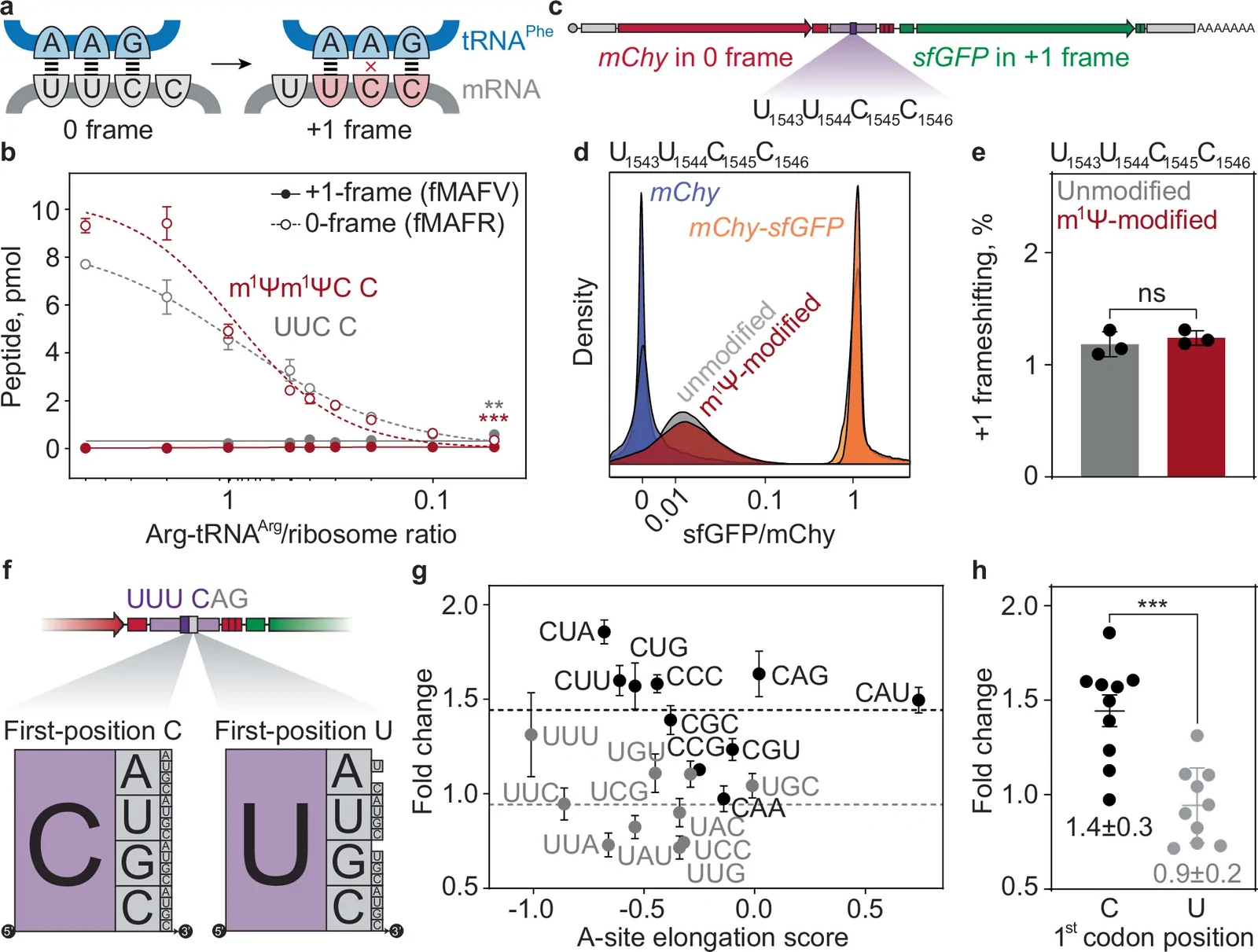
Modulation of m^1^Ψ-induced +1 frameshifting. **a** Non-slippery synonymous UUCC (or m^1^Ψm^1^ΨCC) sequence prevents base-pairing with tRNA^Phe^ (blue) in the +1 frame. **b** Synthesis of 0-frame fMAFR (open circles and dotted line) and +1-frame fMAFV (closed circles and solid line) products during translation of UUCC (gray) and m^1^Ψm^1^ΨCC (maroon) at decreasing Arg-tRNA^Arg^ to ribosome ratio. Shown are mean values with error bars representing SE from *n* = 3 independent biological replicates. Statistical significance by ANOVA test: ***P = 0.0008 (F(1, 4) = 84.08; df = 1); **P = 0.0020 (F(1, 4) = 50.86; df = 1). **c** Schematic of the dual-fluorescence mRNA reporter containing synonymous, non-slippery UUCC substitution. **d** Representative sfGFP/mChy ratio distribution after transfection of HEK293T with mRNA containing only *mChy* (light blue for unmodified; dark blue for m^1^Ψ-modified), *sfGFP* in-frame with *mChy* (*mChy-sfGFP*; light orange for unmodified and dark orange for m^1^Ψ-modified), and mRNA reporters containing the BNT162b2 U_1543_U_1544_C_1545_C_1546_ non-slippery variant (gray for unmodified; maroon for m^1^Ψ-modified). **e** +1 frameshifting on the synonymous non-slippery U_1543_U_1544_C_1545_C_1546_ variant after transfection with unmodified (gray) or m^1^Ψ-modified (maroon) mRNA reporters. Shown are values in individual replicates (black circles) and mean values with error bars representing SE from *n* = 3 biological replicates. ns, *P* = 0.5012 (*t* = 0.7573; df = 3.170), according to two-tailed Welch’s *t*-test. **f** Schematic of the dual-fluorescence mRNA reporter carrying nucleotide substitutions (gray) downstream of the slippery codon U_1543_U_1544_U_1545_ (purple). **g** Change in +1 frameshifting on m^1^Ψ-modified, compared to unmodified, mRNA reporters in HEK293T cells, as compared to the dwell time of the ribosome on the respective A-site codon^55^. Shown are mean values with error bars representing SE from *n* = 3 independent biological replicates. Dashed line shows the mean fold change in +1 frameshifting for codons with first-position C (black) and U (gray). **h** Mean fold change in +1 frameshifting in m^1^Ψ-modified compared to unmodified mRNA for a downstream codon with first-position C (black) or U (gray). Shown are mean values from *n* = 3 independent biological replicates (circles) and mean values (solid line) with error bars representing SE. According to a two-tailed Welch’s *t*-test: ***P = 0.0002 (*t* = 4.802; df = 16.71). Exact values are summarized in Supplementary Table 4. Source data are provided as a Source Data file.

Having established that m^1^Ψ promotes +1 frameshifting through P-site destabilization, we next tested whether this effect can be mitigated by synonymous codon substitutions. Replacing a slippery UUU C motif with synonymous non-slippery UUC C should abolish re-pairing in the +1 frame, because it would require base-pairing of tRNA^Phe^ on a non-cognate codon with an A–C mismatch (Fig. 4a). Indeed, translation of the synonymous non-slippery sequences abolished the synthesis of the +1-frame product in vitro (Fig. 4b), indicating that the slippery motif is essential for +1 frameshifting on m^1^Ψ-modified codons, as previously reported^28^. This suppression was also evident in human cells: substitution of the slippery UUU C sequence with the synonymous non-slippery UUC C (Fig. 4c) abolished the difference between the unmodified and m^1^Ψ-modified mRNA of BNT162b2 (Fig. 4d, e) and between the m^1^Ψ-modified slippery and non-slippery sequences of mRNA-1273 in the dual-fluorescence reporter assay (Supplementary Fig. 11a, b). These findings demonstrate that synonymous replacement of slippery codons effectively suppresses m^1^Ψ-induced +1 frameshifting and provide a potential strategy to improve the fidelity of therapeutic mRNAs.

The effects of the fourth-position nucleotide of the slippery sequence (C vs. U) observed in Fig. 1e prompted us to explore additional strategies to mitigate +1 frameshifting, particularly when synonymous replacement of slippery codons is not feasible due to the risk of deleterious effects of synonymous substitutions on protein folding^54^. We tested the role of the downstream codon, since its 1st nucleotide determines tRNA pairing in the +1-frame codon^50^. We substituted the C_1546_A_1547_G_1548_ codon (Gln498 in BNT162b2 ORF; Fig. 1a) downstream of the U_1543_U_1544_U_1545_ slippery codon with sense codons beginning with C or U and measured +1 frameshifting in human cells (Fig. 4f, Supplementary Fig. 11c and Supplementary Table 4). To control for effects of tRNA availability, we correlated fold change in frameshifting between unmodified and m^1^Ψ-modified mRNAs with ribosome A-site dwell times estimated by ribosome profiling^55^, but found no correlation (Fig. 4g). Instead, codons starting with C significantly increased +1 frameshifting on m^1^Ψ-modified mRNA, whereas codons starting with U showed no change (Fig. 4h & Supplementary Table 4). This effect was specific for the 1st codon position (Supplementary Fig. 11d), consistent with the thermodynamic preference of tRNA^Phe^ for UUC compared to UUU codon^50^. Thus, downstream codon identity modulates m^1^Ψ-induced frameshifting, with codons containing C at the 1st position enhancing slippage, whereas those containing U suppress it.

## Discussion

Our study reveals that incorporation of m^1^Ψ into therapeutic mRNAs increases +1 ribosome frameshifting by destabilizing P-site codon–anticodon interactions, thereby promoting slippage when the A site remains vacant. Using mechanistic dissection, we show that frameshifting does not occur during decoding or translocation, but predominantly through a “hungry” P-site stalling pathway, where delayed delivery of the next cognate tRNA provides a window for slippage. Although the underlying mechanism was demonstrated in the bacterial system and remains to be shown in human translation, we did not observe a correlation between +1 frameshifting and estimated tRNA availability in our reporter system. This apparent discrepancy does not exclude a role for tRNA availability under physiological conditions, where cell type- or tissue-specific tRNA pools, codon usage, or stress-induced changes in tRNA abundance may create conditions that favor frameshifting during translation of vaccine mRNAs. Indeed, lipid nanoparticles are taken up by various skin-residing cells upon injection, including dendritic cells, macrophages/monocytes, fibroblasts, adipocytes, and endothelial cells^8^; antigen-presenting cells undergo extensive transcriptional and translational reprogramming upon antigen uptake and maturation^56,57^, while mRNA translation in cancer is heavily dysregulated^58^. Investigating whether cell type- or state-specific variations in tRNA pools and codon usage determine the extent of m^1^Ψ-induced errors during translation of therapeutic mRNAs will provide additional conceptual guidelines for codon optimization in mRNA vaccine design.

We note that low A-site occupancy (which is the prerequisite for “hungry frameshifting”) may arise not only from the delay in delivery of the cognate aa-tRNA, but may also reflect an unfavorable geometry of the codon exposed in the A site resulting from m^1^Ψ modification of the P-site codon. In fact, recent structural work, albeit obtained with a non-slippery CGm^1^Ψ Am^1^ΨC sequence and two tRNAs bound to the A and P sites^31^, indicates structural rearrangements of the codon–anticodon complex in the P site that would also explain the destabilization of the codon–anticodon complex. Our single-molecule and kinetic analyses demonstrate that m^1^Ψ shifts the peptidyl-tRNA equilibrium from the stable P/P state toward a frameshifting-prone non-canonical state, weakening 0-frame pairing and favoring +1-frame re-pairing, which is thermodynamically more stable. Importantly, we identify codon-specific rules that modulate this process: synonymous replacement of slippery motifs abolishes slippage, and downstream codons beginning with C enhance frameshifting whereas those beginning with U suppress it. Together, these findings not only establish the molecular basis of m^1^Ψ-induced +1 frameshifting, but also point to rational sequence optimization strategies to improve the fidelity of therapeutic mRNAs.

Optimization of the fourth nucleotide of the slippery sequence opens new opportunities for improving mRNA vaccine design. On one hand, rational mitigation of recoding could be achieved by prioritizing synonymous non-slippery sequences, in agreement with previous studies^28^, and avoiding downstream codons with first-position C. On the other hand, deliberate modulation of +1 frameshifting through mRNA sequence design may provide novel therapeutic strategies for personalized cancer vaccines. Recent studies highlight neoantigens generated by translation dysregulation as promising immunotherapeutic targets in cancer, owing to their immunogenicity and tumor specificity^58–63^. Engineered vaccine mRNAs promoting error-prone translation of personalized tumor-specific antigens may diversify the neoantigen pool and broaden immune responses against heterogeneous tumor populations, particularly in tumors with low mutational burden.

Finally, our findings raise broader questions about how epitranscriptomic marks modulate ribosome dynamics to drive recoding events. Beyond m^1^Ψ, other mRNA modifications, such as pseudouridine or 5-methylcytidine, may also modulate ribosome conformational dynamics in ways that favor recoding^23–26^. Our findings also suggest that the effects of clinically relevant mRNA modifications on translation fidelity should be evaluated alongside their effects on immunogenicity and expression, providing an additional criterion for assessing their suitability for therapeutic and vaccine applications. Deciphering these mechanisms will not only illuminate the regulatory networks that govern translation, but also expand the repertoire of clinically relevant mRNA modifications.

## Methods

### Materials

Newly generated materials are available for reuse from the corresponding authors upon request.

### Preparation of mRNAs

The mRNAs for HEK293T transfection and flow cytometry experiments were prepared by in vitro transcription using a DNA template that contained the T7 promoter, 5′ and 3′ UTRs from BNT162b2 or mRNA-1273, and the respective *mChy* and *sfGFP* ORFs. DNA was amplified by polymerase chain reaction (PCR) and transcribed by addition of T7 RNA polymerase (1.6 unit/µl), nucleotide triphosphates (ATP, NU-1010; GTP, NU-1012; CTP, NU1011; and either UTP, NU-1013, or m^1^ΨTP, NU-890; 3 mM each, Jena Bioscience), RNase inhibitor (0.2 units/µl, moloX GmbH), *E. coli* pyrophosphatase (0.005 units/µl, New England Biolabs, M0361) in buffer (40 mM Tris-HCl (Sigma-Aldrich, 252859) pH 7.5, 15 mM MgCl_2_ (Sigma-Aldrich, M9272), 2 mM spermidine (Sigma-Aldrich, S2501), 10 mM NaCl (Sigma-Aldrich, S7653), 10 mM dithiothreitol (Roth, 6908-4)) and incubated at 37 °C for 3 h. The RNA product was purified by fast protein liquid chromatography in buffer (30 mM BisTris (Roth, 9140.3), pH 6, 1 mM EDTA (Merck, 137004)) using a linear gradient of 0.3–1.5 M NaCl, followed by ethanol precipitation. The concentration was determined by UV absorbance at 260 nm. To install the 5′ cap, purified mRNA was mixed with GTP (0.5 mM, New England Biolabs, N2080), S-adenosylmethionine (200 µM, New England Biolabs, B9003), Vaccinia capping enzyme (0.5 units/µl, New England Biolabs, M2080), mRNA cap 2′-O-methyltransferase (2.5 units/µl, New England Biolabs, M0366), Murine RNase Inhibitor (1 U/μl, New England Biolabs, M0314) in capping buffer (New England Biolabs, B2080A) and incubated at 37 °C for 1 h, following the manufacturer’s protocol. The addition of the 3′ poly(A) tail was performed by adding ATP (1 mM, New England Biolabs, P0756) and *E. coli* poly(A) polymerase (0.25 units/µl, New England Biolabs, M0276) to the 5′ capping reaction and incubating at 37 °C for 30 min. The reaction was quenched with EDTA (10 mM, Merck, 137004), and the mRNA was purified using the Monarch Spin RNA Cleanup Kit (New England Biolabs, T2030) following the manufacturer’s protocol. The concentration was determined by UV absorbance at 260 nm.

For the in vitro reconstituted *E. coli* translation assays and smFRET experiments, the following mRNAs were purchased from Integrated DNA Technologies (IDT; mRNAs presented in 5′ → 3′ direction, in bold is the start codon, underlined are the slippery sequences, codons used in translation experiments are separated by hyphens):

Biotin-CAACCUAAAACUUACACACCCGGCAAGGAGGUAAAUA**AUG**-GCA-UUU-CGU-GAUUACCUAA

Biotin-CAACCUAAAACUUACACACCCGGCAAGGAGGUAAAUA**AUG**-GCA-m^1^Ψm^1^Ψm^1^Ψ-CGU-GAUUACCUAA

Biotin-CAACCUAAAACUUACACACCCGGCAAGGAGGUAAAUA**AUG**-GCA-UUC-CGU-GAUUACCUAA

Biotin-CAACCUAAAACUUACACACCCGGCAAGGAGGUAAAUA**AUG**-GCA-m^1^Ψm^1^ΨC-CGU-GAUUACCUAA

CAACCUAAAACUUACACACCCGGCAAGGAGGUAAAUA**AUG**-GCA-UUU-GUG-AUUACCUAA

Biotin-CAACCUAAAACUUACACACCCGGCAAGGAGGUAAAUA**AUG**-GCA-AAA-AAG-GUG-AUUACCUAA

Biotin-CAACCUAAAACUUACACACCCGGCAAGGAGGUAAAUA**AUG**-GCm^1^Ψ-m^1^Ψm^1^ΨC-GUG-AUUACCUAA

Biotin-CAACCUAAAACUUACACACCCGGCAAGGAGGUAAAUA**AUG**-GCU-UUC-GUG-AUUACCUAA

### HEK293T transfection and flow cytometry

200,000–300,000 HEK293T cells (provided by V. Cordes, Max Planck Institute for Multidisciplinary Sciences) were seeded in Dulbecco’s Modified Eagle Medium (DMEM, 4.5 g/l glucose (Gibco, 11995065), 10% fetal bovine serum (Gibco, A3382001)) in 24-well plate and incubated at 37 °C, 5% CO_2_, 95% humidity for 24 h. 5′-capped and 3′-polyadenylated mRNA was mixed with Opti-MEM (Gibco, 31985062) and Lipofectamine^TM^ MessengerMAX^TM^ (Thermo Fisher Scientific, LMRNA008) and incubated at 37 °C, 5% CO_2_, 95% humidity for 24 h. In every experiment, unmodified and m^1^Ψ-modified control mRNAs were included. The cells were trypsinized, resuspended in 1 ml of phosphate-buffered saline (PBS, Gibco, 10010023), and kept on ice. Resuspended cells were filtered, and fluorescence intensities of 100,000 single cells were measured using a BD Aria^TM^ III Cell Sorter flow cytometer. Flow cytometry data compensation, initial analysis, and gating were performed for cells transfected with unmodified and m^1^Ψ-modified mRNAs using the FlowJo^TM^ v10.8 Software (BD Life Sciences, Ashland, OR: Becton, Dickinson and Company; 2023). Single live cells exhibiting mChy and/or sfGFP fluorescence were gated and compensated using the mRNA controls containing a single fluorescent protein and the empty mRNA control to correct for signal overlap between sfGFP and mChy. For each cell, the ratio between corrected sfGFP and mChy fluorescence was calculated using R. Dot plot distribution and calculation of the median of sfGFP/mChy ratio was performed using R package (Eq. 1). The frameshifting frequency of each population was then calculated as the ratio between the median of its sfGFP/mChy ratio distribution to the median of the constitutive frameshifting mRNA control, multiplied by 100 (Eq. 2).

1 $$x={median} (\frac{{sfGFP}}{{mCherry}})$$

2 $${F}_{frameshifting}=\frac{{x}_{sample}}{{x}_{constitutive}}100\%$$

GraphPad Prism 9 software was used for the calculation of statistical significance. GraphPad Prism 9 and ggplot2 were used for the representation of flow cytometry data.

### Preparation of ribosomes, initiation and elongation factors, and tRNAs

*E. coli* 70S ribosomes, initiation and elongation factors IF1, IF2, IF3, EF-Tu, EF-G^39,64^, [^3^H]fMet-tRNA^fMet^, Ala-tRNA^Ala^, [^14^C]Ala-tRNA^Ala^, Lys-tRNA^Lys^, Phe-tRNA^Phe^, [^14^C]Phe-tRNA^Phe^, [^14^C]Arg-tRNA^Arg^ and Val-tRNA^Val^^39,64^ and EF-P^47,48^ were prepared as previously described.

### smFRET sample preparation, data acquisition, and analysis

Microscopy slides and coverslip preparation were performed as described^22,35,51^. L11 expression, purification, Cy3-maleimide (Cytiva, PA23031) labeling and 70S(L11-Cy3) reconstitution were performed as described^51^. Phe-tRNA^Phe^ labeling with Cy5 NHS ester (Cytiva, PA15101) was carried out as described^51^. To form the IC(L11-Cy3), 70S(L11-Cy3) (1 µM) were mixed with IF1, IF2, IF3 (3 µM), GTP (1 mM, Jena Bioscience, NU-1012), 5′-biotinylated mRNA (3 µM, IDT) and [^3^H]fMet-tRNA^fMet^ in TAKM_7_ (50 mM Tris-HCl, 70 mM NH_4_Cl (Supelco, 1011430050), 30 mM KCl (Supelco, 1049361000), 7 mM MgCl_2_, pH 7.5 at 37 °C) and incubated for 30 min at 37 °C. For the TC, EF-Tu (30 µM) was mixed with GTP (1 mM, Jena Bioscience, NU-1012), phosphoenolpyruvate (PEP; 3 mM, Merck, 10108294001) and pyruvate kinase (PK, 1 µM, Roche, 10109045001) and incubated for 30 min at 37 °C, followed by addition of [^14^C]Ala-tRNA^Ala^ (5 µM) and EF-G (2 µM) and incubation for additional 2 min. IC(L11-Cy3) was mixed with TC and incubated for 5 min at 37 °C to generate the POST complex carrying fMet-Ala-tRNA^Ala^ in the P site and an empty A site presenting the UUU or m^1^Ψm^1^Ψm^1^Ψ codon. The POST complex was purified by ultracentrifugation through sucrose cushion (1.1 M) in TAKM_21_ (50 mM Tris-HCl, 70 mM NH_4_Cl, 30 mM KCl, 21 mM MgCl_2_, pH 7.5 at 37 °C) in an Ultima MAX-XP ultracentrifuge (Beckmann Coulter Life Sciences) at 200,000 × *g* for 2 h at 4 °C, dissolved in TAKM_7_ buffer and quantified via scintillation counting of ^3^H and ^14^C radioactivity.

Reaction chambers were incubated with TAKM_7_SP (50 mM Tris-HCl pH 7.5 at 22 °C, 70 mM NH_4_Cl, 30 mM KCl, 7 mM MgCl_2_, 8 mM putrescine (Roth, 4141.2), and 1 mM spermidine, Sigma-Aldrich, S2501) containing BSA (10 mg/ml, Sigma-Aldrich, A4503) and neutravidin (1 μM, Thermo Scientific, 31000) for 5 min at room temperature. Neutravidin was washed by adding the same buffer without neutravidin^22,35^. The respective ribosome complex was added to the reaction chamber and incubated for 2 min at room temperature. For smFRET experiments monitoring decoding, POST(L11-Cy3) complexes were diluted to 1 nM in TAKM_7_SP and were immobilized on the coverslip. For smFRET experiments monitoring P-site tRNA dynamics, purified POST(L11-Cy3) (0.1 µM) was mixed with TC (EF-Tu–Phe-tRNA^Phe^(Cy5)–GTP, 0.5 µM) and EF-G (2 µM), incubated for 5 min at 37 °C to form the POST(L11-Cy3) carrying fMet-Ala-Phe-tRNA^Phe^(Cy5) in the P site, diluted to 1 nM in TAKM_7_SP, and immobilized on the coverslip. Imaging started after addition of TAKM_7_SP containing protocatechuic acid (2.5 mM, Sigma-Aldrich, 37580), *Pseudomonas* protocatechuate-3,4-dioxygenase (50 nM, Merck, 9029-47-4), 6-hydroxy-2,5,7,8-tetramethylchromane-2-carboxylic acid (2 mM, Sigma-Aldrich, 238813), and methylviologen (1 mM, Sigma-Aldrich, 856177). For smFRET experiments monitoring decoding, the imaging buffer was additionally supplemented with GTP (1 mM, Jena Bioscience, NU-1012) and TC with EF-Tu(wt)– or EF-Tu(H84A)–GTP–Phe-tRNA^Phe^(Cy5) (5 nM).

TIRF imaging was performed at 22 °C on an IX 81 inverted microscope using a PLAPON 60 × 1.45 numerical aperture objective (Olympus) and collected using xCellence imaging software (Olympus). Cy3 was excited using a 561 nm solid-state laser operated at 25 mW and images were recorded with an electron-multiplying CCD camera (CCD-C9100-13, Hamamatsu) at a rate of 30.3 frames/s. Color channels were separated by projecting donor and acceptor emission onto different parts of the CCD chip using an image splitter (dual view micro imager DV2, Photometrics), filter specifications HQ 605/40, HQ 680/30 (Chroma Technology). Fluorescence time courses for Cy3 and Cy5 were extracted using custom-made MATLAB (MathWorks) software according to published protocols^22,35^. Cy3 bleed-through into the Cy5 channel was corrected using an experimentally determined coefficient of 0.13. FRET efficiency (E_FRET_)was calculated as the ratio of the measured emission fluorescence intensities (F. I.; Eq. 3).

3 $${E}_{{FRET}}=\frac{{{FI}}_{{Cy}5}}{{{FI}}_{{Cy}5}+{{FI}}_{{Cy}3}}\,$$

A semi-automated algorithm (MATLAB) was used to select single fluorophores, based on fluorescence-intensity criteria characteristic of single donor–acceptor complexes and single-step photobleaching. smFRET trajectories were fitted by a Hidden Markov model using the vbFRET software package (http://vbfret.sourceforge.net/)^65^ to generate the idealized trajectories, and only trajectories displaying the expected anticorrelation of donor and acceptor fluorescence intensities were retained for further analysis. Trajectories were truncated to remove photobleaching and photoblinking events. Histograms compiling FRET traces for a given complex were fitted to a sum of Gaussian functions using MATLAB code with an unconstrained nonlinear minimization procedure (fminsearch, MATLAB, R2011b). The number of states was established by selecting the number of Gaussian components that adequately described the FRET distributions, minimized residuals, and yielded reproducible state parameters across independent datasets. Progressively increasing the number of components did not substantially improve the fit and resulted in unstable or poorly resolved parameters, indicating overfitting. The number of FRET states was further validated by Hidden Markov modeling in individual trajectories and dwell-time distribution analysis. Two-dimensional contour plots were generated from raw time-resolved FRET trajectories using custom-made software^51^. Dwell times of different FRET states were calculated from idealized trajectories. FRET changes in idealized trajectories that were smaller than the s.d. of the Gaussian distribution of the FRET states were not considered transitions because they could not be distinguished from the noise. The cumulative dwell-time distribution was fitted to an exponential function (Eq. 4) to calculate the decay rate *k*_*fit*_ (Eq. 5).

4 $$y={y}_{0}+A{e}^{-t/\tau }\,$$

5 $$\,{k}_{{fit}}=1/\tau \,$$

Rates were corrected (Eq. 6)^35^.

6 $${k}_{{corrected}}={k}_{{fit}}-{k}_{{FRET}}-1/T$$

where *k*_*fit*_ is the rate of the exponential decay function of the cumulative dwell-time distribution, *k*_*FRET*_ is the rate of loss of FRET signal under the described imaging conditions, 0.03 ± 0.01 s^−1^, and *T* is the observation time, 33 s. GraphPad Prism 9 software was used for the exponential function fit and representation of smFRET data.

### In vitro reconstituted translation from *E. coli*

ICs were prepared in TAKM_7_ (50 mM Tris-HCl, 70 mM NH_4_Cl, 30 mM KCl, 7 mM MgCl_2_, pH 7.5 at 37 °C) by mixing 70S ribosomes (1 µM) with a 3-fold excess of IF1, IF2, IF3, mRNA, [^3^H]fMet-tRNA^fMet^ and GTP, incubation at 37 °C for 30 min and purification by ultracentrifugation through sucrose cushion (1.1 M) in TAKM_21_ (50 mM Tris-HCl pH 7.5 at 37 °C, 70 mM NH_4_Cl, 30 mM KCl, 21 mM MgCl_2_) using an Ultima MAX-XP ultracentrifuge (Beckmann Coulter Life Sciences) at 200,000 × *g* at 4 °C for 2 h. The pellet was dissolved in TAKM_7_, and IC concentration was determined based on ^3^H scintillation counting. Ternary complex was prepared in TAKM_7_ (50 mM Tris-HCl, 70 mM NH_4_Cl, 30 mM KCl, 7 mM MgCl_2_, pH 7.5 at 37 °C) buffer by mixing EF-Tu (3-fold over tRNAs), GTP (1 mM, Jena Bioscience, NU-1012), phosphoenolpyruvate (PEP; 3 mM) and pyruvate kinase (PK, 1 µM, Roche, 10109045001) and incubation at 37 °C for 15 min. A mixture of Ala-tRNA^Ala^ (5-fold over IC), Phe-tRNA^Phe^ (5-fold over IC), [^14^C]Arg-tRNA^Arg^ (5-fold over IC), Val-tRNA^Val^ (5-fold over IC), and EF-G (wt or EF-G(Q507D), 2 μM) was added, and the sample was incubated at 37 °C for 2 min. Purified IC (0.2 μM) was added to the ternary complex, and translation was carried out for 5 min at 37 °C. The reaction was quenched in KOH (0.5 M, Supelco, 1.05033) for 30 min at 37 °C and neutralized with glacial acetic acid (10%, Supelco, 100066). Translation products were separated via reverse-phase high-performance liquid chromatography (HPLC, Separation Module Waters 2695) on an RP-8 column (Supelco, LiChrospher® WP300, 5 µm, 836339) using a 0–65% acetonitrile (Supelco, 100029) linear gradient in 0.1% trifluoroacetic acid (Sigma-Aldrich, 302031). Fractions were mixed with Ultima Gold^TM^ XR scintillation cocktail (Revvity, 6013119), and ^3^H and ^14^C radioactivity was measured by scintillation counting (Liquid Scintillation Analyzer, PerkinElmer, Tri-Carb 3110TR). GraphPad Prism 9 software was used for the calculation of statistical significance, hyperbolic function fit (Hill’s equation, Eq. 7), and representation of the data.

7 $$y=\frac{{B}_{\max }{x}^{h}}{{K}_{d}^{h}+{x}^{h}}\,$$

### Peptidyl-tRNA drop-off assay

TC was prepared as described above using Ala-tRNA^Ala^ or [^14^C]Ala-tRNA^Ala^ (5-fold over IC), [^14^C]Phe-tRNA^Phe^ (5-fold over IC), and EF-G (wt, 2 µM). Purified IC (0.2 μM) was added to TC, and the sample was incubated for 2 min at 37 °C to form the POST complex. After 2 min, aliquots of the POST complex (5 pmol) at the indicated time points were spread on 0.45-μm nitrocellulose filters (Sartorius, 11306-25-N). The filter was washed with TAKM_7_, dissolved in 1 ml soluene®−350 (Revvity, 6003038), and mixed with Ultima Gold^TM^ XR scintillation cocktail (Revvity, 6013119), and ^3^H and ^14^C radioactivity was measured by scintillation counting (PerkinElmer, Tri-Carb 3110TR). GraphPad Prism 9 software was used for the calculation of statistical significance, single (Eq. 4) and two-phase (Eq. 8) exponential function fits, and representation of the data.

8 $$y={Plateau}+{A}_{{fast}}{e}^{-{k}_{{fast}}x}+{A}_{{slow}}{e}^{-{k}_{{slow}}x}$$

where Plateau is the baseline value y approaches as x goes to infinity, A_fast_ and A_slow_ are the amplitudes that the fast and slow phase contribute, *k*_*fast*_ and *k*_*slow*_ are the rate constants for fast and slow decays.

### Reporting summary

Further information on research design is available in the Nature Portfolio Reporting Summary linked to this article.

## Supplementary information

Supplementary Information

Reporting Summary

Transparent Peer Review file

## Source data

Source Data

## Data Availability

The data generated in this study are provided in the Supplementary Information and Source Data file. The flow cytometry data generated in this study are available in the figshare database under doi:10.6084/m9.figshare.33120299 (https://figshare.com/s/d71493167137da4a4709). Source data are provided with this paper.
