# Supplementary material for: Recoding by N1-methylpseudouridine guides rational mRNA vaccine design
Source: Nat Commun. 2026 Sep 28;17:10029. doi: 10.1038/s41467-026-77796-3 (PMC13620113; doi:10.1038/s41467-026-77796-3)
Supplement: Supplementary file 1 [file 41467_2026_77796_MOESM1_ESM.pdf]

Supplementary Information for

Recoding by N^1-methylpseudouridine guides rational mRNA vaccine design

Panagiotis Poulis[1,2]✉, Giovanni Robecchi[1,2], Arina O. Kurochkina[1], Michele Felletti[1] & Marina V. Rodnina[1]✉

[1]Department of Physical Biochemistry, Max Planck Institute for Multidisciplinary Sciences, Am Fassberg 11, 37077 Göttingen, Germany

[2]These authors contributed equally: Panagiotis Poulis, Giovanni Robecchi

✉corresponding authors; rodnina@mpinat.mpg.de, panagiotis.poulis@mpinat.mpg.de

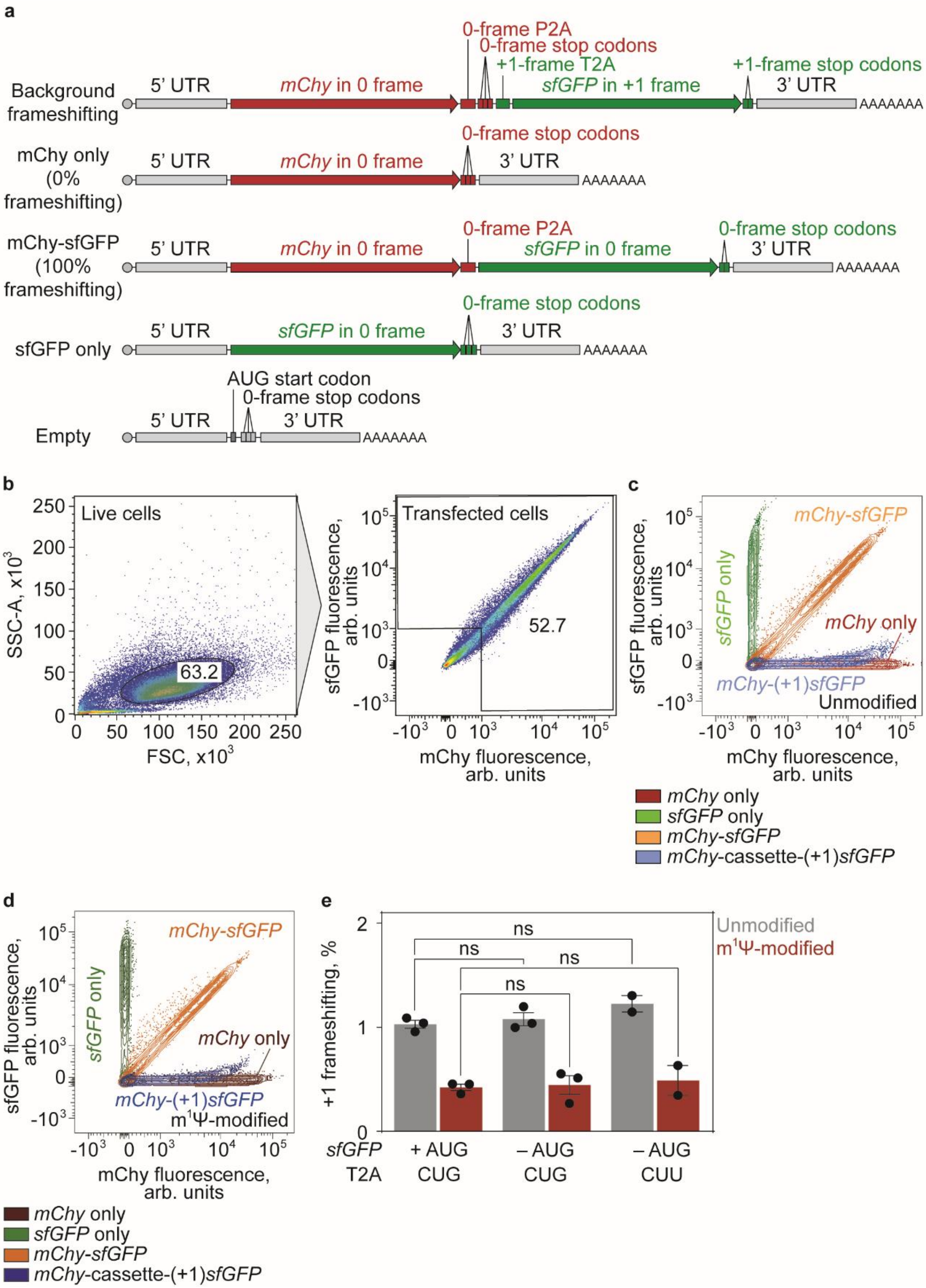
a
0-frame P2A
0-frame stop codons
+1-frame T2A
+1-frame stop codons
Background frameshifting
5' UTR
mChy in 0 frame
sfGFP in +1 frame
3' UTR
AAAAAAA
mChy only (0% frameshifting)
0-frame stop codons
mChy-sfGFP (100% frameshifting)
sfGFP in 0 frame
sfGFP only
AUG start codon
Empty
b
Live cells
63.2
SSC-A, x10^3
FSC, x10^3
Transfected cells
52.7
sfGFP fluorescence, arb. units
mChy fluorescence, arb. units
c
mChy-sfGFP
sfGFP only
mChy only
mChy-(+1)sfGFP
Unmodified
mChy-cassette-(+1)sfGFP
d
m1Ψ-modified
e
+1 frameshifting, %
ns
Unmodified
m1Ψ-modified
sfGFP
T2A
+ AUG
CUG
– AUG
CUG
– AUG
CUU

◄**Supplementary Figure 1. Analysis of m^{1}Ψ-induced +1 frameshifting by flow cytometry.** (a) Schematic representation of mRNA reporters used to transfect HEK293T cells. In the mRNA designed to detect the extent of background +1 frameshifting, the slippery cassette was omitted. The mRNA that represents 0% frameshifting contains only the *mChy* ORF, while the mRNA that provides 100% +1 frameshifting efficiency contains *sfGFP* in-frame with *mChy* in the absence of stop codons between the two ORFs. (b) Representative flow cytometry dot plots after transfection of HEK293T with mRNA reporters. Numbers indicate the percentage of cells within the area demarcated by solid lines. (c) Representative flow cytometry contour plot after transfection of HEK293T cells with unmodified mRNA containing only *mChy* (maroon), only *sfGFP* (green), *sfGFP* in-frame to *mChy* (*mChy-sfGFP*; orange) and construct containing slippery cassette between *mChy* and +1-frame *sfGFP* (blue). Each dot represents single event through the flow cytometer. (d) Same as (c) for m^{1}Ψ-modified mRNA reporters. Only *mChy*, dark maroon; only *sfGFP*, dark green; *mChy-sfGFP*, dark orange; construct containing slippery cassette between *mChy* and +1-frame *sfGFP*, dark blue. (e) +1 frameshifting (%) after transfection of HEK293T cells with unmodified (grey) and m^{1}Ψ-modified (maroon) background +1 frameshifting mRNA reporter, after removing the AUG start codon of *sfGFP* and additionally mutating the near-cognate CUG start codon of T2A to the synonymous non-cognate CUU. Shown are values in individual replicates (black circles) and mean values with error bars representing standard error (SE) from n=2 (–AUG, CUU) and n=3 (+AUG, CUG; –AUG, CUG) independent biological replicates. ns, statistically non-significant difference in +1 frameshifting compared to respective +1 frameshifting values in the presence of *sfGFP* AUG and CUG in T2A, according to two-tailed Welch's t test. + AUG/CUG vs. – AUG/CUG, P= 0.5479 (t= 0.6671; degrees of freedom (df)= 3.338) for unmodified mRNA; P= 0.8300 (t= 0.2382; df= 2.482) for m^{1}Ψ-modified mRNA. + AUG/CUG vs. – AUG/CUU, P= 0.1978 (t= 2.231; df= 1.484) for unmodified mRNA; P= 0.7252 (t= 0.4506; df= 1.055) for m^{1}Ψ-modified mRNA. Source data are provided as a Source Data file.

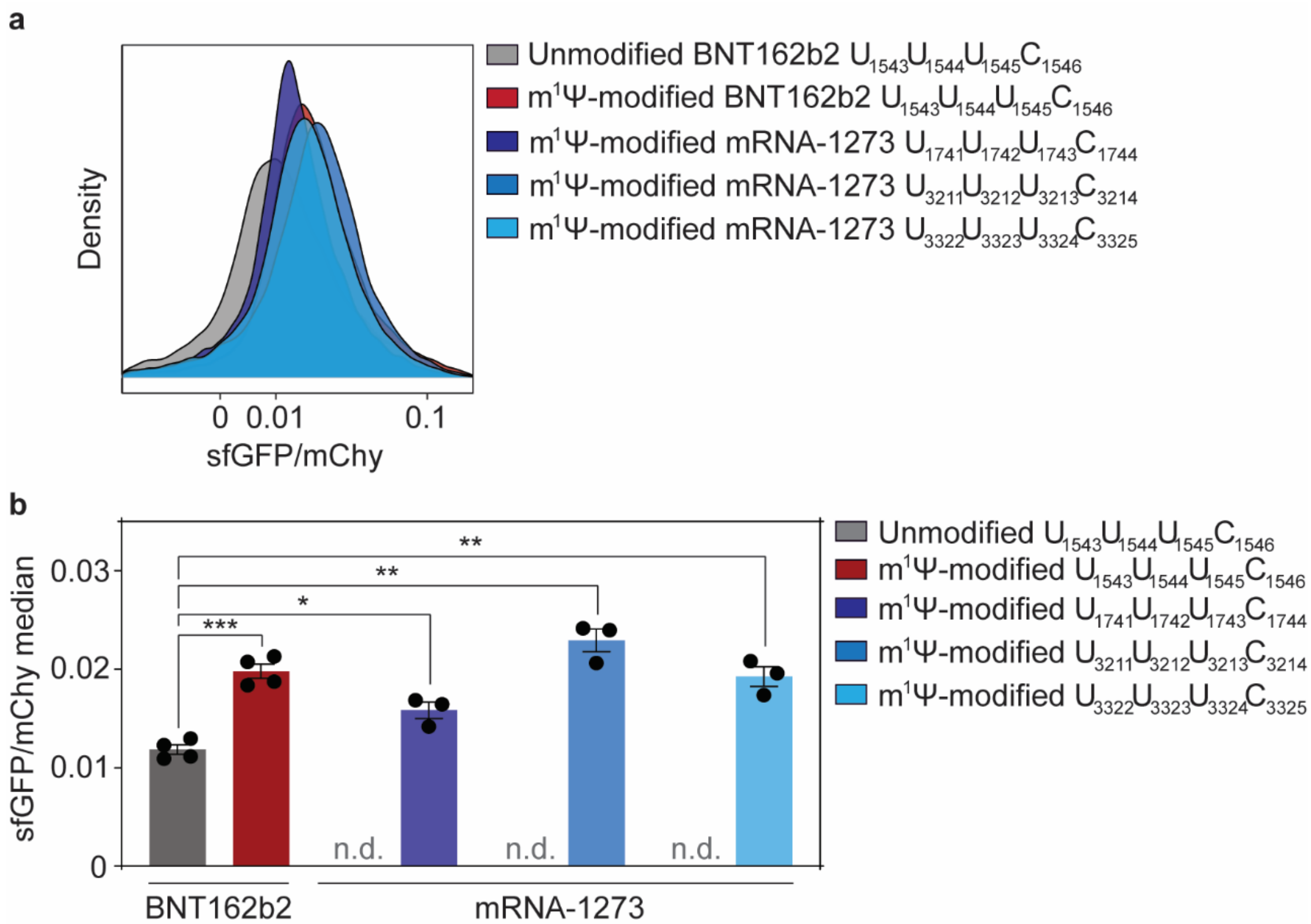

Supplementary Figure 2. $m^1\Psi$-induced +1 frameshifting on slippery sequences of mRNA-1273 reporter mRNAs in human cells. (a) Representative sfGFP/mChy ratio distribution after transfection of HEK293T with mRNA reporters containing the $U_{1543}U_{1544}U_{1545}C_{1546}$ slippery sequence of BNT162b2 (grey for unmodified; maroon for $m^1\Psi$-modified) and the three UUU C slippery sequences of mRNA-1273 (blue shades) between mChy and +1-frame sfGFP. Numbering of the nucleotides is based on the original sequence of BNT162b2 and mRNA-1273. (b) Median of sfGFP/mChy ratio distribution measured by flow cytometry after transfection with mRNA reporters carrying the slippery $U_{1543}U_{1544}U_{1545}C_{1546}$ of BNT62b2 (grey for unmodified, maroon for $m^1\Psi$-modified) and the three slippery UUU C sequences of mRNA-1273 (sfGFP/mChy ratio distribution median cannot be determined for unmodified reporter mRNA due to low expression, n.d.; blue shades for $m^1\Psi$-modified). Shown are values in individual replicates (black circles) and mean values with error bars representing SE from n=3 (mRNA-1273) and n=4 (BNT162b2) independent biological replicates. Asterisks represent statistically significant difference, according to two-tailed Welch's t test. Statistical significance from left to right: ***, *P*=0.0002 (t=9.088; degrees of freedom (df)=5.229); *, *P*=0.0212 (t=4.126; df=3.319); **, *P*=0.0043 (t=8.929; df=2.729); **, *P*=0.0074 (t=6.647; df=2.937). Source data are provided as a Source Data file.

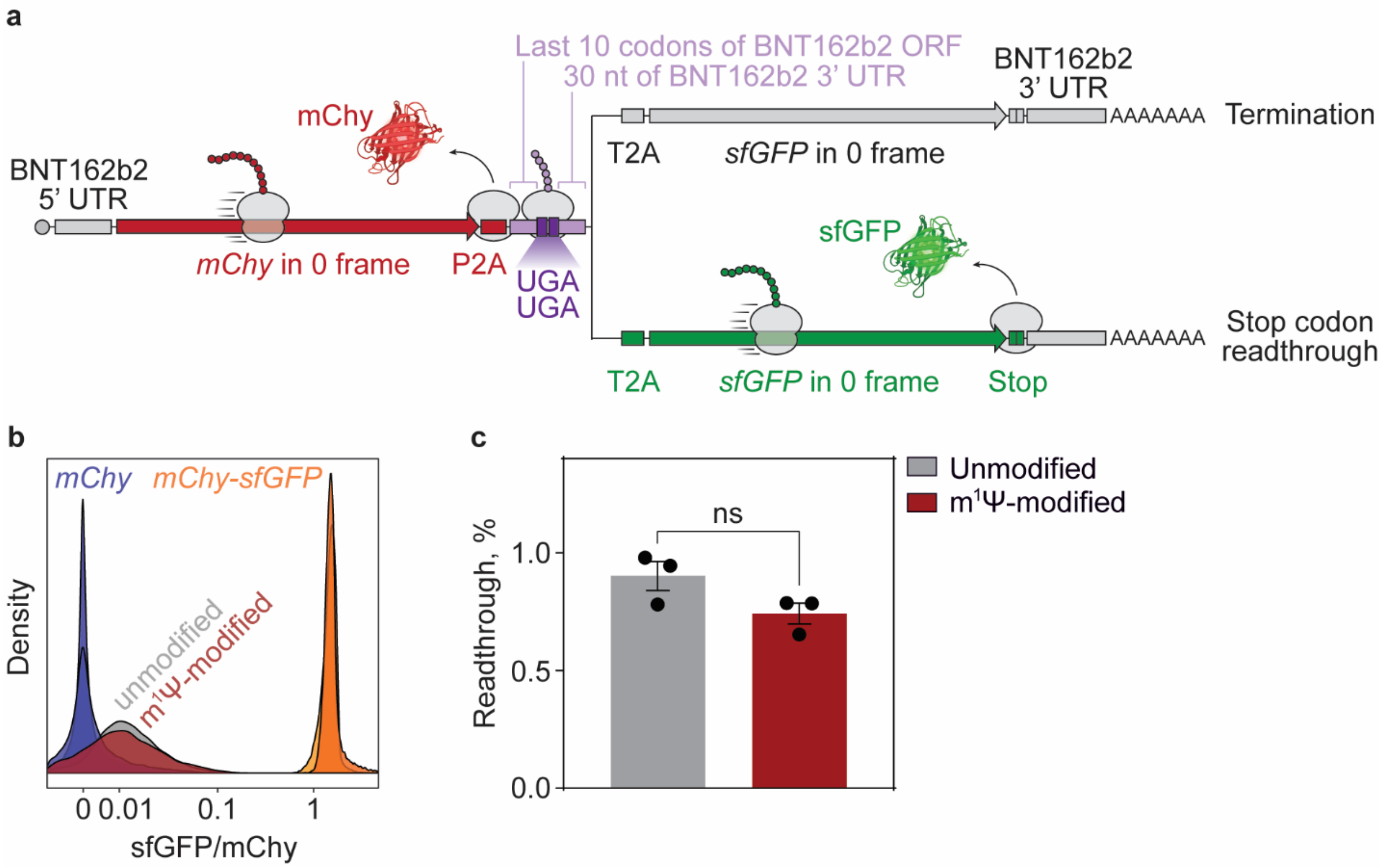

Supplementary Figure 3. m¹Ψ does not drive stop codon readthrough ≥1%. (a) Schematic of the dual fluorescence mRNA reporter to monitor stop codon readthrough *in vivo*. Upon canonical termination on the two consecutive UGA stop codons (purple) of the BNT162b2 cassette (lilac), only mChy (red) is produced (upper branch). In case of readthrough, translation resumes to produce sfGFP (green; lower branch). Part of the figure was created in BioRender. Poulis, P. (2026) https://BioRender.com/cyswms8. (b) Representative sfGFP/mChy ratio distribution on mRNA reporters containing only *mChy* (light blue for unmodified, dark blue for m¹Ψ-modified), *sfGFP* in-frame to *mChy* (*mChy-sfGFP*; light orange for unmodified, dark orange for m¹Ψ-modified) and containing the BNT162b2 stop cassette between *mChy* and *sfGFP* (grey for unmodified, maroon for m¹Ψ-modified). (c) Stop codon readthrough (%) on the two consecutive UGA stop codons of BNT162b2, measured by flow cytometry after transfection with unmodified (grey) or m¹Ψ-modified (maroon) mRNA reporters. Shown are values in individual replicates (black circles) and mean values with error bars representing SE from n=3 independent biological replicates. ns, statistically non-significant difference, P=0.1087 (t=2.117; df=3.630), according to two-tailed Welch's t test. Exact values are summarized in Supplementary Table 1. Source data are provided as a Source Data file.

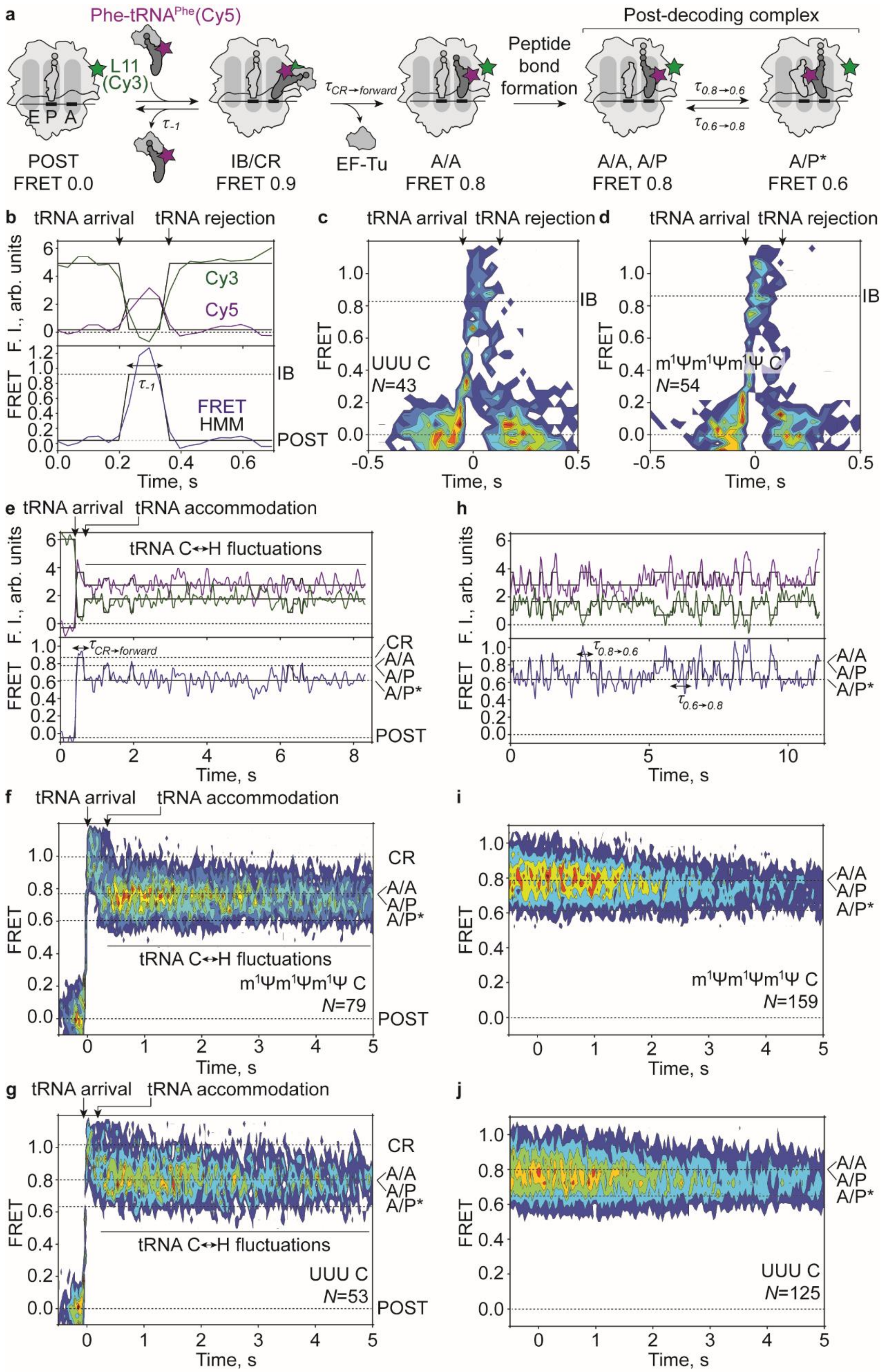
a
Phe-tRNA^Phe(Cy5)
Post-decoding complex
L11 (Cy3)
E P A
τ-1
τCR→forward
Peptide bond formation
τ0.8→0.6
τ0.6→0.8
POST FRET 0.0
IB/CR FRET 0.9
EF-Tu
A/A FRET 0.8
A/A, A/P FRET 0.8
A/P* FRET 0.6
b
tRNA arrival
tRNA rejection
Cy3
Cy5
FRET
HMM
IB
POST
F. I., arb. units
Time, s
c
UUU C
N=43
d
m1Ψm1Ψm1Ψ C
N=54
e
tRNA accommodation
tRNA C↔H fluctuations
CR
A/A
A/P
A/P*
h
f
N=79
i
N=159
g
N=53
j
N=125

◄**Supplementary Figure 4. Decoding of unmodified and m^{1}Ψ-modified codons monitored by smFRET.** (a) Schematic of the elemental steps during decoding monitored by single-molecule FRET. After addition of TC containing Phe-tRNA^{Phe}(Cy5) (purple), FRET increases due to initial binding (IB) and codon recognition (CR) and then decreases upon tRNA accommodation in the A site ($\tau_{CR\rightarrow forward}$; A/A conformation). After peptide bond formation, peptidyl-tRNA^{Phe}(Cy5) interconverts between A/A, A/P and A/P* conformations. (b) Representative smFRET time courses of Cy3 (green) and Cy5 (purple) fluorescence intensity (F. I.), calculated FRET (blue), and hidden Markov model fit (HMM, black) corresponding to tRNA rejection after initial binding (IB). (c) Contour plot showing FRET distribution of traces corresponding to tRNA rejection after IB, during decoding of UUUC codon. Post-synchronization was performed at the time point with FRET>0.0. Shown are compiled data from n=3 independent biological replicates. FRET 0.81 ± 0.08 (IB; FRET is shown as mean and standard deviation of the center, μ, of the Gaussian distribution from n=3 independent biological replicates). *N*, number of traces. (d) Same as (c) during decoding of m^{1}Ψm^{1}Ψm^{1}ΨC codon. FRET 0.86 ± 0.05 (IB). (e) Representative smFRET time courses of Cy3 (green) and Cy5 (purple) F. I., FRET (blue), and HMM (black) corresponding to the complete decoding reaction from codon reading (CR) to interconversion between A/A, A/P and A/P* states. (f) Contour plot showing FRET distribution of traces corresponding to the complete decoding reaction, during decoding of UUUC codon. Shown are compiled data from n=3 independent biological replicates. (g) Same as (f) during decoding of m^{1}Ψm^{1}Ψm^{1}ΨC codon. (h) Representative smFRET time courses of Cy3 (green) and Cy5 (purple) F. I., FRET (blue), and HMM (black) corresponding to post-decoding interconversions between A/A, A/P and A/P* states.(i) Contour plot showing FRET distribution of traces corresponding to post-decoding tRNA interconversions on the UUUC codon. Shown are compiled data from n=3 independent biological replicates. FRET 0.82 ± 0.09 (A/A, A/P) and FRET 0.66 ± 0.05 (A/P*). (j) Same as (i) on the m^{1}Ψm^{1}Ψm^{1}ΨC codon. FRET 0.79 ± 0.03 (A/A, A/P) and 0.62 ± 0.05 (A/P*). Source data are provided as a Source Data file.

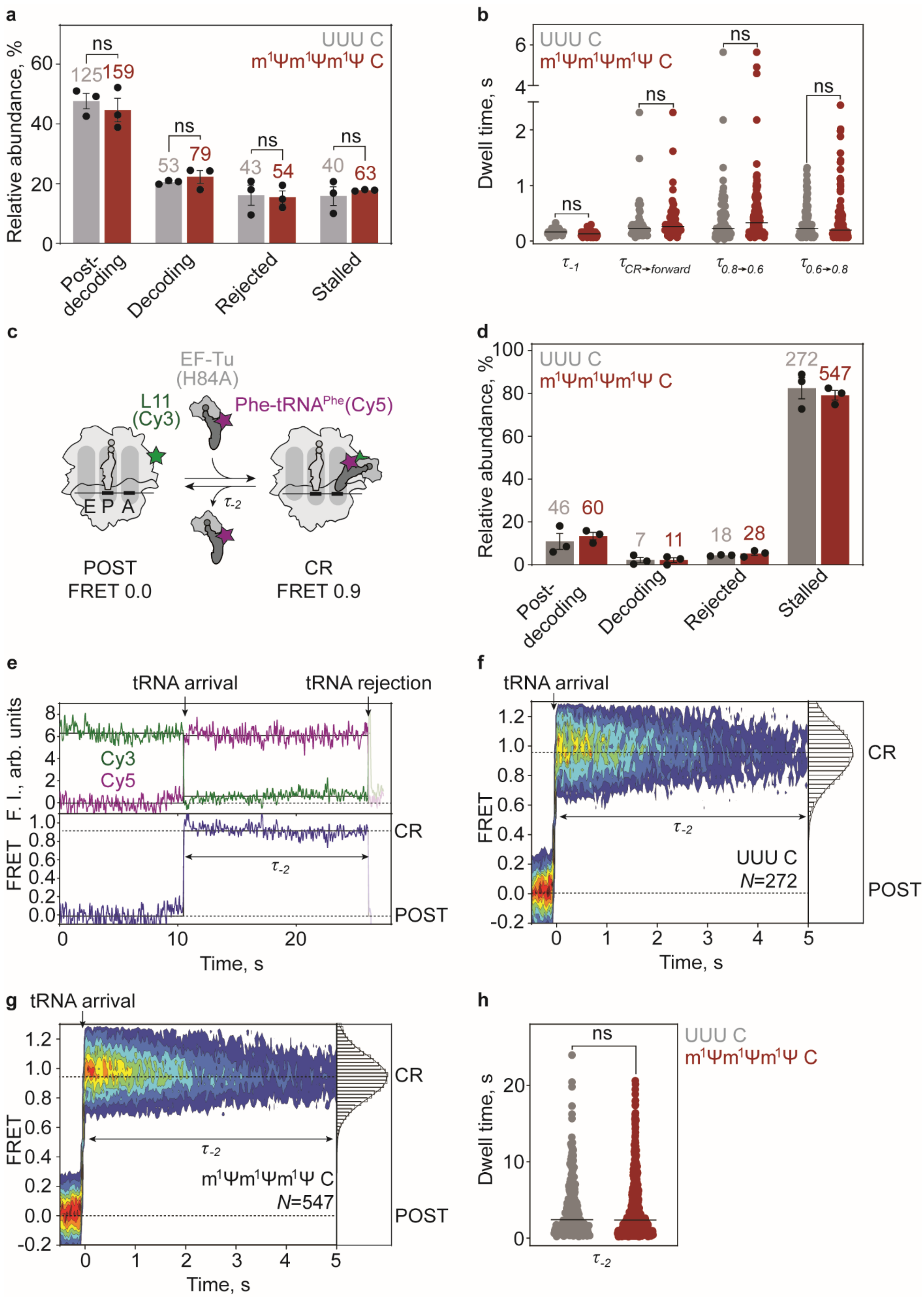

a
Relative abundance, %
UUU C
m¹Ψm¹Ψm¹Ψ C
ns
125
159
53
79
43
54
40
63
Post-decoding
Decoding
Rejected
Stalled
b
Dwell time, s
τ-1
τCR→forward
τ0.8→0.6
τ0.6→0.8
c
EF-Tu (H84A)
L11 (Cy3)
Phe-tRNAPhe(Cy5)
E P A
τ-2
POST FRET 0.0
CR FRET 0.9
d
272
547
46
60
7
11
18
28
e
tRNA arrival
tRNA rejection
F. I., arb. units
Cy3
Cy5
FRET
CR
POST
Time, s
f
UUU C N=272
g
m¹Ψm¹Ψm¹Ψ C N=547
h

◄**Supplementary Figure 5. Decoding kinetics of unmodified and m^{1}Ψ-modified codons.** (a) Relative abundance of the different classes of traces during decoding of UUUC (grey) and m^{1}Ψm^{1}Ψm^{1}ΨC (maroon) in the presence of EF-Tu(wt). Shown are values of individual replicates (black circles) and mean values with error bars representing SE from n=3 independent biological replicates. Asterisks represent statistically significant differences according to two-tailed Welch's t test. ns, statistically non-significant difference (from left to right: P=0.5690 (t=0.6284; df=3.440); P=0.5017 (t=0.8045; df=2.110); P=0.8707 (t=0.1754; df=3.405); P=0.6016 (t=0.6139; df=2.008). Number of traces (N) is indicated. (b) Dwell time distribution of key steps during decoding of UUUC (grey) and m^{1}Ψm^{1}Ψm^{1}ΨC (maroon). Shown are values of individual traces (circles) and median (solid line) of data from n=3 independent biological replicates. ns, according to Kolmogorov-Smirnov test (from left to right: P=0.2542; P=0.6195; P=0.0965; P=0.9076). (c) Schematic of the smFRET assay to monitor CR state in the presence of EF-Tu(H84A). (d) Relative abundance of the different classes of traces during decoding of UUUC (grey) and m^{1}Ψm^{1}Ψm^{1}ΨC (maroon) in the presence of EF-Tu(H84A). Shown are values of individual replicates (circles) and mean values with error bars representing SE from n=3 independent biological replicates. (e) Representative smFRET time courses of Cy3 (green) and Cy5 (purple) F. I., calculated FRET (blue) and HMM (black) corresponding to stalling at the CR state.(f) Contour plot showing FRET distribution of traces stalled at the CR state, during decoding of UUUC codon in the presence of EF-Tu(H84A). Histogram at the right shows distribution of FRET values after post-synchronization. Shown are compiled data from n=3 independent biological replicates. FRET 0.94 ± 0.02 (CR). (g) Same as (f) during decoding of m^{1}Ψm^{1}Ψm^{1}ΨC codon in the presence of EF-Tu(H84A). FRET 0.95 ± 0.03 (CR). (h) Dwell time distribution of CR state during decoding of UUUC (grey) and m^{1}Ψm^{1}Ψm^{1}ΨC (maroon) in the presence of EF-Tu(H84A). Shown are values of individual traces (circles) and median (solid line) of compiled data from n=3 independent biological replicates. ns, according to Kolmogorov-Smirnov test, P=0.8993. Exact values are summarized in Supplementary Table 2. Source data are provided as a Source Data file.

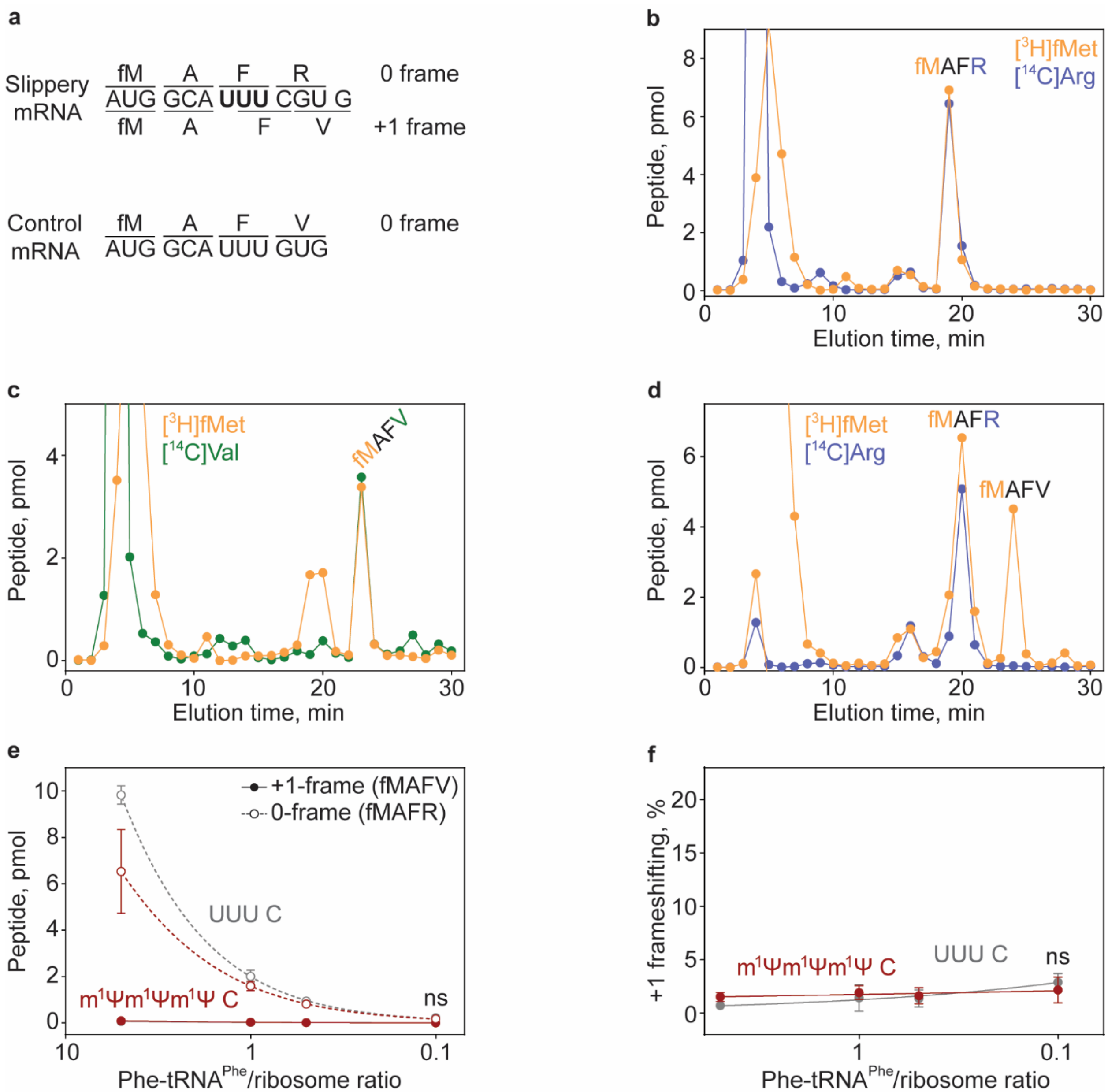

Supplementary Figure 6. Analysis of 0- and +1-frame peptide products. (a) mRNA sequences used in this study. The slippery mRNA contains a UUU C or m¹Ψm¹Ψm¹Ψ C motif, which encodes for fMet-Ala-Phe-Arg (fMAFR) in 0 frame and fMet-Ala-Phe-Val (fMAFV) in +1 frame. The control mRNA encodes for fMet-Ala-Phe-Val (fMAFV) in 0 frame. (b) HPLC chromatogram showing [^{3}H]fMet (orange) and [^{14}C]Arg (blue) amount (pmol) during translation of the slippery mRNA in the absence of Val-tRNA^{Val}, identifying the elution time of the fMAFR peptide. (c) HPLC chromatogram showing [^{3}H]fMet (orange) and [^{14}C]Val (green) amounts during translation of the control mRNA, identifying the elution time of the fMAFV peptide. (d) HPLC chromatogram showing [^{3}H]fMet (orange) and [^{14}C]Arg (blue) amounts during translation of the

slippery mRNA in the presence of Val-tRNA^{Val}, identifying the elution of fMAFR and fMAFV peptides. (e) Synthesis of 0-frame fMAFR (open circles and dotted line) and +1-frame fMAFV (closed circles and solid line) products during translation of UUU C (grey) and m^{1}Ψm^{1}Ψm^{1}Ψ C (maroon) sequences at decreasing Phe-tRNA^{Phe} to ribosome ratio. Shown are mean values with error bars representing SE from n=3 independent biological replicates. ns, statistically non-significant difference in +1-frame peptide synthesis, according to two-way repeated measures ANOVA test, P=0.6401 (F(1, 4)=0.2551; df=1). Lines are hyperbolic function (Methods). (f) Lack of +1 frameshifting (%, calculated from the data in panel (e)) during translation of the slippery UUU C (grey) and m^{1}Ψm^{1}Ψm^{1}Ψ C (maroon) codons at decreasing Phe-tRNA^{Phe} to ribosome ratio. Shown are mean values with error bars representing SE from n=3 independent biological replicates. ns, statistically non-significant difference according to two-way repeated measures ANOVA, P=0.8332 (F(1, 4)=0.05048; df=1). Lines are hyperbolic function (Methods). Source data are provided as a Source Data file.

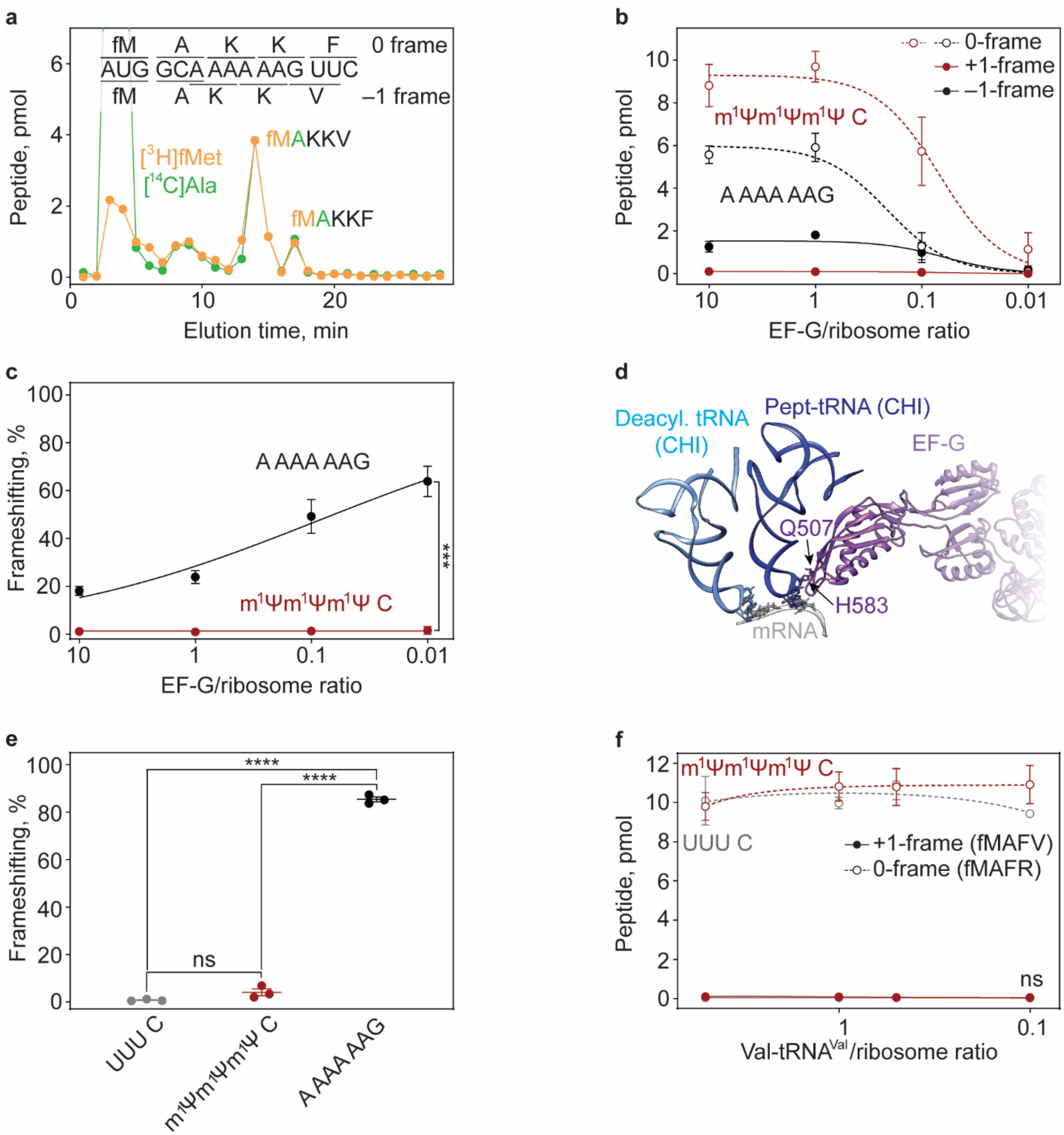

Supplementary Figure 7. Ribosome frameshifting routes during translocation on $m^1\Psi$-modified slippery codons. (a) HPLC peptide analysis of translation products of the –1 frameshifting-prone A AAA AAG sequence using [^{3}H]fMet (orange) and [^{14}C]Ala (green) scintillation counting. (b) Synthesis of 0-frame peptides fMAFR and fMAKKF (open circles and dotted lines) and the respective +1-frame fMAFV (maroon closed circles and solid line) and –1-frame fMAKKV (black closed circles and solid line) peptides during translation of mRNAs containing $m^1\Psi m^1\Psi m^1\Psi$ C (maroon) and A AAA AAG (black) sequence, respectively, at

decreasing EF-G to ribosome ratio. Shown are mean values with error bars representing SE from n=3 independent biological replicates. Lines are hyperbolic function. (c) Frameshifting efficiency (%) during translation of m¹Ψm¹Ψm¹Ψ C (maroon) and A AAA AAG (black) at decreasing EF-G to ribosome ratio. Shown are mean values with error bars representing SE from n=3 independent biological replicates. Asterisk represents the statistically significant difference in frameshifting using two-way repeated measures ANOVA test, *** P=0.0001 (F(1, 4)=213.5; df=1). Lines are hyperbolic function. (d) Structure of ribosome complexes containing pept-$tRNA^{Phe}$ (blue) and deacylated $tRNA^{fMet}$ (light blue) at the CHI state. EF-G (purple) contacts the codon-anticodon duplex via the key residues Q507 and H583 (arrows; PDB 7PJY). (e) Frameshifting during translation of UUU C (grey), m¹Ψm¹Ψm¹Ψ C (maroon) and A AAA AAG (black) slippery sequences induced by EF-G(Q507D). Shown are values of individual replicates (black circles) and mean values (solid line) with error bars representing SE from n=3 independent biological replicates. Asterisks represent statistically significant difference according to two-tailed Welch's t test, **** P<0.0001 (t=80.13; df=2.152 for UUU C; t=45.77; df=3.630 for m¹Ψm¹Ψm¹Ψ C; ns, non-significant, P=0.1479 (t=2.257; df=2.079). (f) Synthesis of 0-frame fMAFR (open circles and dotted line) and +1-frame fMAFV (closed circles and solid line) products during translation of UUU C (grey) and m¹Ψm¹Ψm¹Ψ C (maroon) sequences at decreasing Val-$tRNA^{Val}$ to ribosome ratio. Shown are mean values with error bars representing SE from n=3 independent biological replicates. ns, not significant difference according to two-way repeated measures ANOVA test, P=0.4616 (F(1, 4)=0.6616; df=1). Lines are hyperbolic function. Source data are provided as a Source Data file.

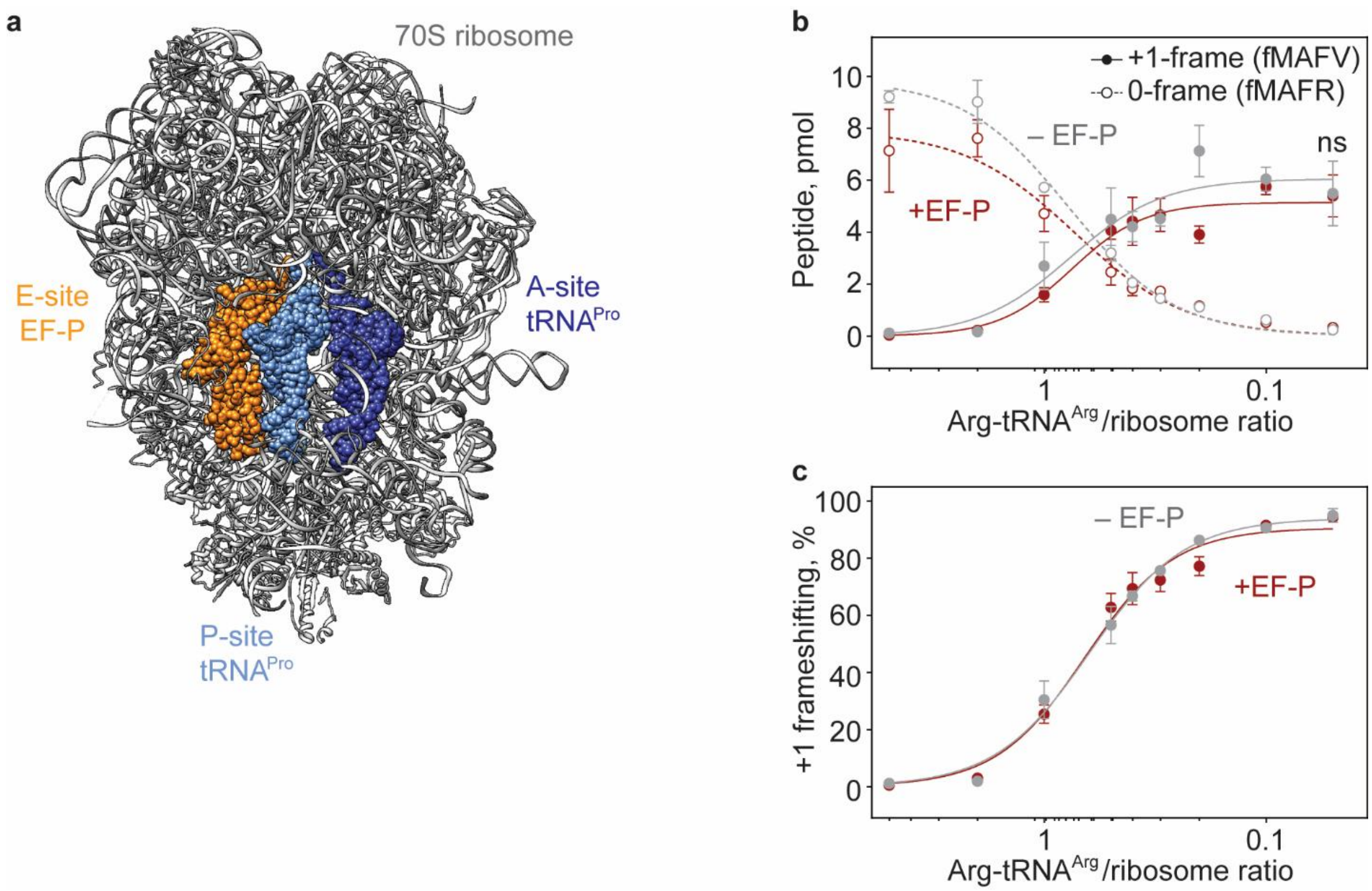

Supplementary Figure 8. No effect of EF-P on m^{1}Ψ-induced +1 frameshifting. (a) Cryo-EM structure of 70S ribosome complexes (grey) containing tRNA^{Pro} in the A (dark blue) and P (light blue) site and EF-P (orange) at the E site (PDB 6ENJ)[1]. (b) Synthesis of 0-frame fMAFR (open circles) and +1-frame fMAFV (closed circles) products during translation of m^{1}Ψm^{1}Ψm^{1}Ψ C at decreasing Arg-tRNA^{Arg} to ribosome ratio in the presence (maroon) or absence (grey) of EF-P. Shown are mean values with error bars representing SE from n=3 independent biological replicates. ns, not significant difference according to two-way repeated measures ANOVA test, P=0.3257 ($F(1, 4)$=1.253; df=1). Lines are hyperbolic function (Methods). (c) +1 frameshifting efficiency (%, calculated from the data in panel (b)) during translation of m^{1}Ψm^{1}Ψm^{1}Ψ C at decreasing Arg-tRNA^{Arg} to ribosome ratio, in the presence (maroon) or absence (grey) of EF-P. Shown are mean values with error bars representing SE from n=3 independent biological replicates. Lines are hyperbolic function (Methods). Source data are provided as a Source Data file.

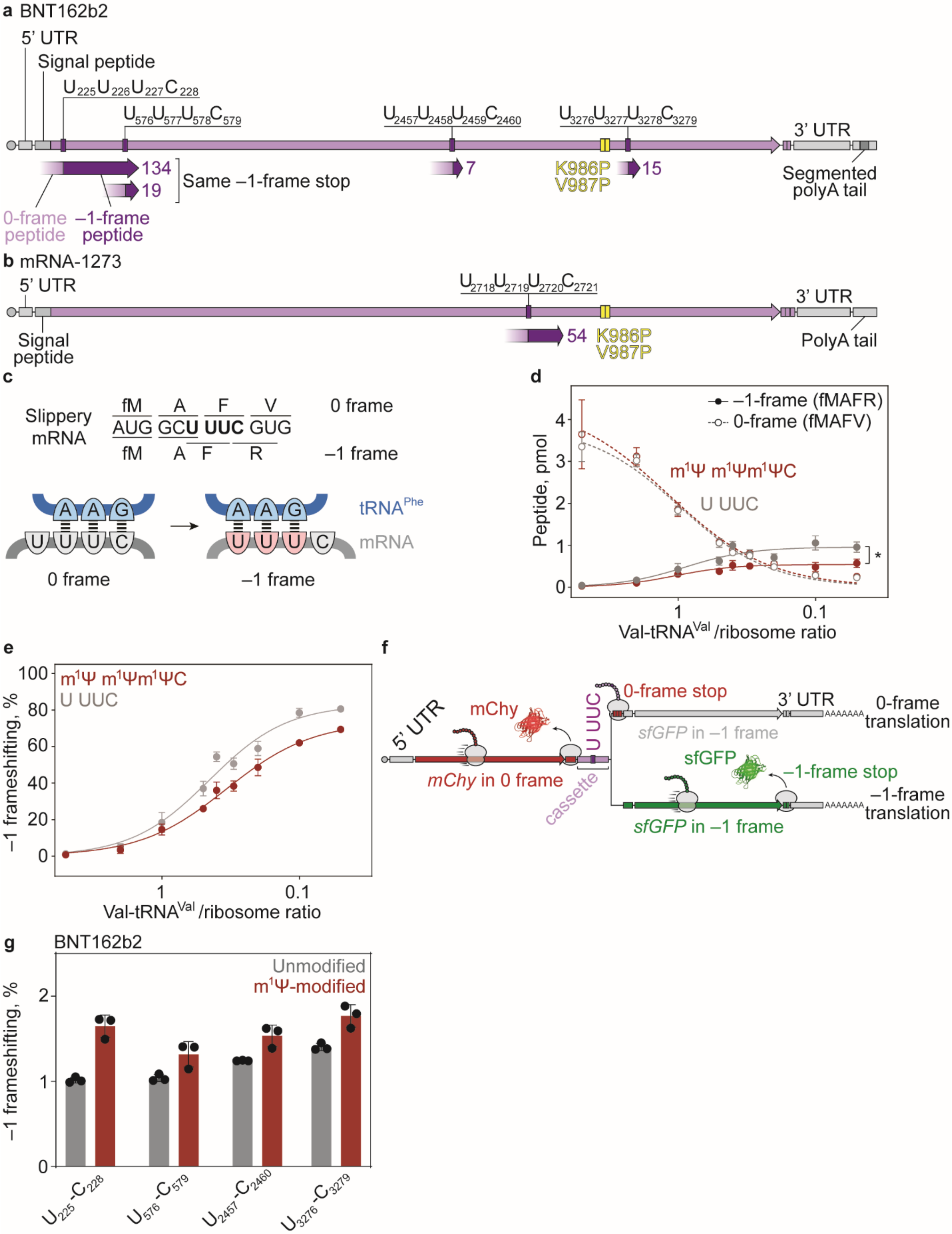
a BNT162b2
5' UTR
Signal peptide
U225U226U227C228
U576U577U578C579
U2457U2458U2459C2460
U3276U3277U3278C3279
3' UTR
134
19
Same –1-frame stop
7
K986P
V987P
15
Segmented polyA tail
0-frame peptide
–1-frame peptide
b mRNA-1273
5' UTR
U2718U2719U2720C2721
3' UTR
54
K986P
V987P
Signal peptide
PolyA tail
c
Slippery mRNA
fM A F V
AUG GCU UUC GUG
0 frame
fM A F R
–1 frame
tRNAPhe
mRNA
0 frame
–1 frame
d
–1-frame (fMAFR)
0-frame (fMAFV)
m1Ψ m1Ψm1ΨC
U UUC
Peptide, pmol
Val-tRNAVal /ribosome ratio
e
m1Ψ m1Ψm1ΨC
U UUC
–1 frameshifting, %
Val-tRNAVal /ribosome ratio
f
5' UTR
mChy
mChy in 0 frame
U UUC
cassette
0-frame stop
3' UTR
0-frame translation
sfGFP in –1 frame
sfGFP
–1-frame stop
–1-frame translation
sfGFP in –1 frame
g BNT162b2
Unmodified
m1Ψ-modified
–1 frameshifting, %
U225-C228
U576-C579
U2457-C2460
U3276-C3279

◄**Supplementary Figure 9. No effect of m^{1}Ψ on –1 frameshifting.** (a, b) Schematics of the BNT162b2 (a) and mRNA-1273 (b) vaccine mRNAs, in which the putative –1 frameshifting-prone slippery sequences (purple squares) may, upon –1 frameshifting, produce –1-frame peptides (purple arrows). Numbers in purple indicate the length in amino acid residues (aa) of the –1-frame peptide extensions up to the first –1-frame stop codon. (c) Coding sequence of mRNA carrying the slippery U UUC (or m^{1}Ψ m^{1}Ψm^{1}ΨC, bold) sequence that allows base pairing with tRNA^{Phe} anticodon (blue) in –1 frame (red). (d) Synthesis of 0-frame fMAFV (open circles and dotted line) and –1-frame fMAFR (closed circles and solid line) products during translation of U UUC (grey) and m^{1}Ψ m^{1}Ψm^{1}ΨC (maroon) sequences at decreasing Val-tRNA^{Val} to ribosome ratio. Shown are mean values with error bars representing SE from n=3 independent biological replicates. Asterisk represents statistically significant difference according to two-way repeated measures ANOVA test, * P=0.0176 (F(1, 4)=15.18; df=1). Lines are hyperbolic function (Methods). (e) –1 frameshifting efficiency (calculated from the data in panel (d)) during translation of U UUC (grey) and m^{1}Ψ m^{1}Ψm^{1}ΨC (maroon) sequences at decreasing Val-tRNA^{Val} to ribosome ratio. Shown are mean values with error bars representing SE from n=3 independent biological replicates. Lines are hyperbolic function. (f) Schematic of the dual fluorescence mRNA reporter to monitor +1 frameshifting *in vivo*. In the absence of +1 frameshifting on the slippery sequence (purple) of the BNT162b2 or mRNA-1273 cassette (lilac), translation of *mChy* (red) terminates at 0-frame stop codons (red boxes; upper branch). Upon +1 frameshifting, the 0-frame stop codons are omitted and translation resumes in +1 frame to produce sfGFP (green; lower branch). Part of the figure was created in BioRender. Poulis, P. (2026) https://BioRender.com/cyswms8. (g) –1 frameshifting on the slippery motifs of BNT162b2 measured by flow cytometry after transfection with unmodified (grey) and m^{1}Ψ-modified (maroon) mRNA reporters. Shown are values in individual replicates (black circles) and mean values with error bars representing standard error (SE) from n=3 independent biological replicates. Source data are provided as a Source Data file.

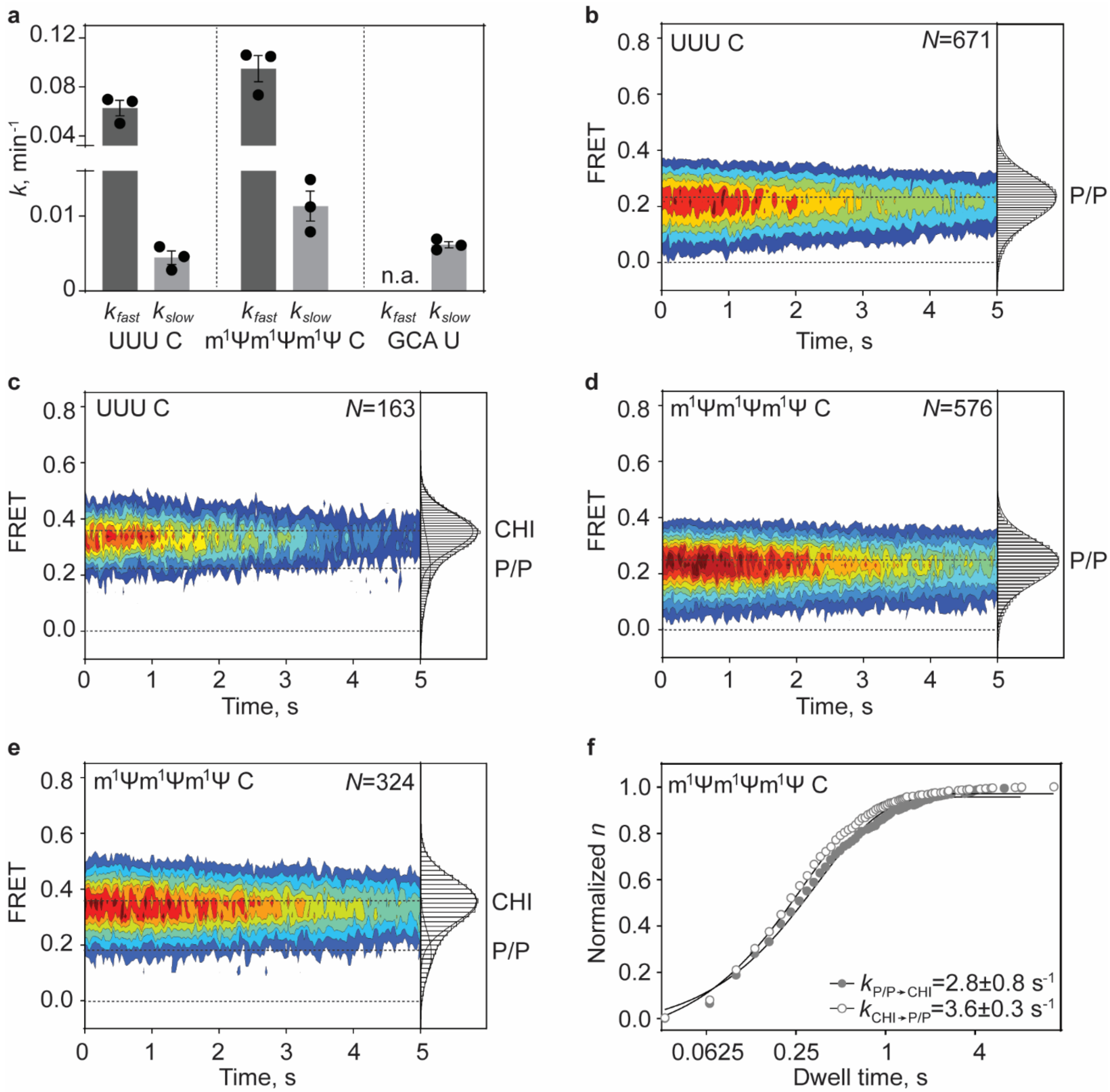

Supplementary Figure 10. Kinetics of P-site peptidyl-tRNA^{Phe} fluctuations monitored by smFRET. (a) Drop-off rates (k, min^{-1}) of the P-site peptidyl-tRNA from the UUU C (left), m^{1}Ψm^{1}Ψm^{1}Ψ C (middle) and GCA U (right) codons. Shown are values in individual replicates (black circles) and mean with error bars representing SE from n=3 independent biological replicates. n.a., k_{fast} is not applicable because the data for GCA U follow single exponential function. Exact values are summarized in Supplementary Table 3. (b) Contour plot showing FRET distribution of traces showing stable FRET 0.2 (P/P) state on the UUU C codon. Histogram at the right shows distribution of FRET values. Shown are data compiled from n=3 independent biological replicates, each with 10 technical replicates. FRET 0.23 ± 0.005 (P/P). N, number of

traces. (c) Same as (a) for the $m^1\Psi m^1\Psi m^1\Psi$ C codon. FRET 0.24 ± 0.005 (P/P). (d) Contour plot showing FRET distribution of traces showing fluctuations between FRET 0.2 (P/P) and FRET 0.35 (CHI) states on UUU C. Histogram at the right shows distribution of FRET values. Shown are data (grey circles) compiled from n=3 independent biological replicates, each with n=10 technical replicates. FRET 0.35 ± 0.005 (CHI) and 0.18 ± 0.01 (P/P). *N*, number of traces. (e) Same for the $m^1\Psi m^1\Psi m^1\Psi$ C codon. FRET 0.36 ± 0.01 (CHI) and 0.19 ± 0.02 (P/P). (f) Cumulative dwell time histogram of the transition from FRET 0.19 ± 0.02 (P/P) and FRET 0.36 ± 0.01 (CHI) state (closed circles) and the transition from FRET 0.36 ± 0.01 (CHI) to FRET 0.19 ± 0.02 (P/P) state (open circles) on $m^1\Psi m^1\Psi m^1\Psi$ C codon. Shown are data and the rate constant as mean values with SD from n=3 biological replicates, each with n=10 technical replicates. Lines are single exponential decay function (Methods). Exact values summarized in Supplementary Table 3. Source data are provided as a Source Data file.

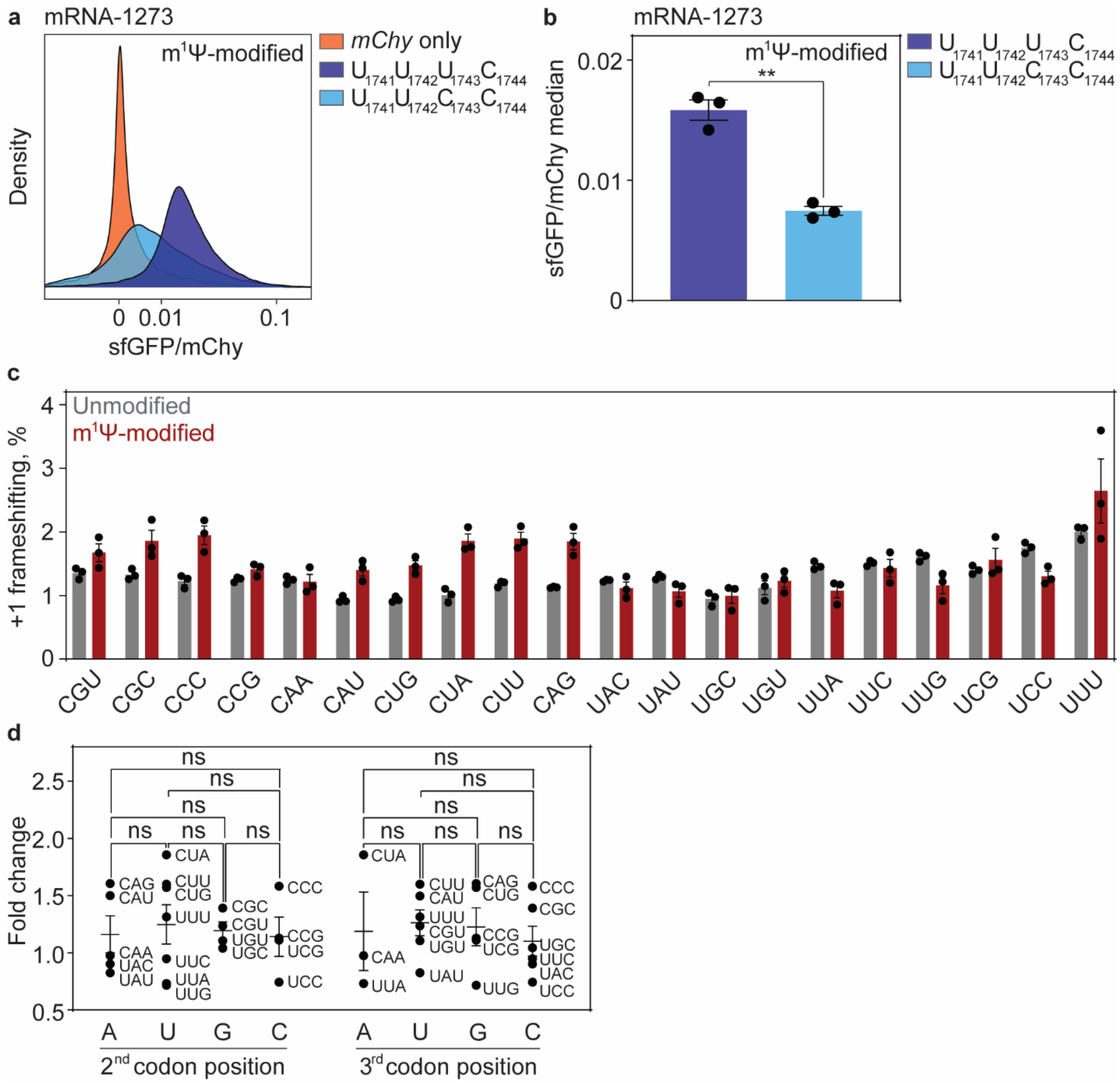

Supplementary Figure 11. Codon context on $m^1\Psi$-induced +1 frameshifting. (a) Representative sfGFP/mChy ratio distribution after transfection of HEK293T with $m^1\Psi$-modified mRNA reporters containing only *mChy* (orange) and $m^1\Psi$-modified mRNA reporters containing the slippery $U_{1741}U_{1742}U_{1743}C_{1744}$ sequence of mRNA-1273 (dark blue) and the synonymous non-slippery $U_{1741}U_{1742}C_{1743}C_{1744}$ sequence (light blue) between mChy and +1-frame sfGFP. (b) Median of sfGFP/mChy ratio distribution on the slippery $U_{1741}U_{1742}U_{1743}C_{1744}$ and the synonymous non-slippery $U_{1741}U_{1742}C_{1743}C_{1744}$ of mRNA-1273 measured by flow cytometry after transfection of HEK293T cells with $m^1\Psi$-modified mRNA reporters. Shown are values in individual replicates (black circles) and mean values with error bars representing SE from 3 biological replicates.

Numbering of the nucleotides is based on the original sequence of mRNA-1273. Asterisk represents the statistically significant difference according to two-tailed Welch's t test, ** P=0.0039 (t=9.147; df=2.744). (c) +1 frameshifting efficiency (%) after transfection of HEK293T cells with unmodified (grey) and $m^1\Psi$-modified mRNA (maroon) carrying different downstream 0-frame codons with first-position C or U. Shown are values in individual replicates (black circles) and mean with error bars representing SE from n=3 independent biological replicates. Exact values are summarized in Supplementary Table 4. (d) Fold change in +1 frameshifting during translation of $m^1\Psi$-modified mRNA reporters, compared to unmodified mRNA, carrying downstream 0-frame codons bearing second- or third-position A, U, G or C. Shown are mean values of fold-change in +1 frameshifting for each codon (circles) from n=3 independent biological replicates and mean values (solid line) with error bars representing SE. ns, statistically non-significant difference according to Brown-Forsythe and Welch ANOVA test. 2nd codon position: A vs. U, P=0.9992 (t=0.3644; df=9.792); A vs. G, P>0.9999 (t=0.1728; df=5.633); A vs. C, P>0.9999 (t=0.08660; df=6.728); U vs. G, P=0.9997 (t=0.2923; df=8.015); U vs. C, P=0.9975 (t=0.4389; df=8.006); G vs. C, P=0.9997 (t=0.2737; df=4.167); 3rd codon position: A vs. U, P=0.9999 (t=0.2073; df=2.455); A vs. G, P>0.9999 (t=0.1021; df=2.963); A vs. C, P=0.9999 (t=0.2349; df=2.602); U vs. G, P>0.9999 (t=0.1791; df=7.366); U vs. C, P=0.9139 (t=0.9286; df=9.819); G vs. C, P=0.9881 (t=0.5926; df=8.038). Exact values are summarized in Supplementary Table 4. Source data are provided as a Source Data file.

Supplementary Tables

Supplementary Table 1. Frameshifting and stop codon readthrough after transfection of HEK293T cells with unmodified and $m^1\Psi$-modified mRNA reporters containing BNT162b2 sequences.

**+1 frameshifting-prone slippery sequences**[a]	**+1 frameshifting, %**[b]		**Fold change**[c]
	**Unmodified**	**$m^1\Psi$-modified**	
$U_{766}U_{767}U_{768}\,C_{769}$	1.02±0.05	1.5±0.2	1.5±0.3
$U_{1543}U_{1544}U_{1545}\,C_{1546}$	1.12±0.02	1.8±0.2	1.6±0.2
$U_{1981}U_{1982}U_{1983}\,C_{1984}$	1.6±0.3	2.7±0.5	1.7±0.2
$U_{3178}U_{3179}U_{3180}\,U_{3181}$	1.0±0.1	1.0±0.2	1.07±0.06
$U_{3238}U_{3239}U_{3240}\,C_{3241}$	0.82±0.02	2.6±0.2	3.2±0.2
$U_{3319}U_{3320}U_{3321}\,C_{3322}$	1.7±0.3	1.47±0.08	0.9±0.1
Background +1 frameshifting	1.03±0.05	0.42±0.04	0.41±0.05
**Termination sequence**	**Stop codon readthrough, %**[b]		**Fold change**[c]
	**Unmodified**	**$m^1\Psi$-modified**	
BNT162b2 stop site UGA UGA	0.90±0.09	0.74±0.06	0.82±0.02

[a]Nucleotide numbering is based on the original sequences of the BNT162b2 (Fig. 1a).

[b]Shown are mean values and SD from n=3 independent biological replicates (Fig. 1d, e & Supplementary Fig. 3c).

[c]Fold change is calculated as the ratio of frameshifting or stop codon readthrough efficiency in $m^1\Psi$-modified mRNA reporters to that in unmodified mRNA reporters and is shown as mean values and SD from n=3 independent biological replicates (Fig. 1d, e & Supplementary Fig. 3c).

Supplementary Table 2. smFRET kinetics during decoding of UUU C and $m^1\Psi m^1\Psi m^1\Psi$ C codons.

Codon	UUU C		$m^1\Psi m^1\Psi m^1\Psi$ C	
**Classes of traces**	**EF-Tu(wt)**	**EF-Tu(H84A)**	**EF-Tu(wt)**	**EF-Tu(H84A)**
Rejected, %[a]	16±6	5±1	15±3	4.4±0.3
Decoding, %[a]	20±1	2±2	22±3	2±2
Post-decoding, %[a]	48±4	13±2	45±6	11±5
Stalled, %[a]	16±5	79±3	17.7±0.2	82±7
**Dwell time, τ**	**UUU C**		**$m^1\Psi m^1\Psi m^1\Psi$ C**	
τ_{-1}[b], s (n)[c]	0.17±0.01 (45)		0.12±0.05 (55)	
$\tau_{CR \rightarrow forward}$[b], s ($n$)[c]	0.21±0.05 (45)		0.3±0.1 (74)	
τ_{-2}[d], s^{-1} (n)[c]	2.4±0.2 (272)		2.5±0.2 (547)	
$\tau_{0.8 \rightarrow 0.6}$[b], s ($n$)[c]	0.4±0.2 (94)		0.34±0.01 (139)	
$\tau_{0.6 \rightarrow 0.8}$[b], s ($n$)[c]	0.24±0.03 (91)		0.21±0.01 (136)	

[a]Shown are mean values with SD from n=3 independent biological replicates (Supplementary Fig. 5a, d).

[b]Dwell times were calculated from the experiments with EF-Tu(wt) (Supplementary Fig. 4 & 5b). Shown are mean values of the medians of the dwell time distributions and SD from n=3 independent biological replicates.

[c]n, number of transitions.

[d]τ_{-2} was calculated from the experiments with EF-Tu(H84A) (Supplementary Fig. 5e-h). Shown are mean values of the medians of the dwell time distributions and SD from n=3 independent biological replicates.

Supplementary Table 3. Peptidyl-tRNA drop-off rates and smFRET kinetics of the peptidyl-$tRNA^{Phe}$(Cy5) in the P site.

**Drop-off rates of peptidyl-$tRNA^{Phe}$ from the P site**		
**Drop-off rate**	**UUU C**	**$m^1\Psi m^1\Psi m^1\Psi$ C**
k_{fast}[a], min^{-1}	0.063±0.009	0.095±0.015
k_{slow}[a], min^{-1}	0.004±0.001	0.011±0.003
**Drop-off rate of peptidyl-$tRNA^{Ala}$ from the P site**		
k_{off}[a], min^{-1}	0.006±0.001	
**smFRET kinetics between peptidyl-$tRNA^{Phe}$(Cy5) conformations in the P site**		
**Fraction of smFRET traces**		
**Classes smFRET traces**	**UUU C**	**$m^1\Psi m^1\Psi m^1\Psi$ C**
Static P/P, %[a] (*N*)[b]	81±6 (671)	63±10 (576)
Dynamic, %[a] (*N*)[b]	19±6 (163)	37±10 (324)
**Relative abundance of FRET states**		
**FRET state**	**UUU C**	**$m^1\Psi m^1\Psi m^1\Psi$ C**
FRET 0.2 (P/P), %[a]	85±6	70±8
FRET 0.4 (CHI), %[a]	15±6	30±8
**Transition rates between FRET 0.2 and 0.4 states**		
**Transition rate**	**UUU C**	**$m^1\Psi m^1\Psi m^1\Psi$ C**
$k_{P/P\rightarrow CHI}$[a, c], s^{-1} (*n*)[d]	-[e] (97)	2.8±0.8 (570)
$k_{CHI\rightarrow P/P}$[a, c], s^{-1} (*n*)[d]	-[e] (102)	3.6±0.3 (642)

[a] Shown are mean values with SD from n=3 independent biological replicates.

[b]*N*, number of traces

[c]Shown are rates corrected according to the equation $k_{corrected}=k_{fit}-k_{FRET}-1/T$, where k_{fit} is the rate of the single exponential decay function, k_{FRET} is the rate of loss of FRET signal under the described imaging conditions (Methods), 0.03±0.01 s^{-1} [2], and *T* is the observation time window, 33 s.

[d]*n*, number of transitions

[e]Number of transitions in the individual datasets was too small to fit with exponential decay function.

Supplementary Table 4. +1 frameshifting efficiency after transfection of HEK293T cells with unmodified and $m^1\Psi$-modified mRNA reporters.

Downstream codon[a]	+1 frameshifting, %[b]		Fold change[c]
	Unmodified	$m^1\Psi$-modified	
CGU	1.35±0.07	1.7±0.2	1.24±0.08
CGC	1.33±0.07	1.9±0.2	1.4±0.1
CCG	1.25±0.03	1.41±0.08	1.13±0.04
CCC	1.23±0.08	1.9±0.2	1.58±0.07
CAA	1.24±0.05	1.2±0.2	0.97±0.09
CAU	0.93±0.04	1.4±0.1	1.5±0.1
CUG	0.94±0.04	1.5±0.1	1.6±0.2
CUA	1.00±0.09	1.9±0.2	1.86±0.09
CUU	1.18±0.04	1.9±0.1	1.6±0.1
UAC	1.24±0.01	1.1±0.1	0.9±0.1
UAU	1.29±0.03	1.1±0.1	0.82±0.09
UCC	1.75±0.07	1.3±0.1	0.74±0.04
UGC	0.95±0.09	1.0±0.2	1.04±0.09
UGU	1.1±0.2	1.2±0.1	1.1±0.1
UUA	1.47±0.05	1.1±0.2	0.73±0.09
UUC	1.51±0.04	1.4±0.2	0.9±0.1
UUG	1.61±0.06	1.2±0.2	0.72±0.09
UCG	1.40±0.06	1.6±0.3	1.1±0.1
UUU	2.00±0.09	2.6±0.7	1.3±0.3

[a]Codon substitutions in the codon downstream to the $U_{1543}U_{1544}U_{1545}$ of the BNT162b2 mRNA (Fig. 1a).

[b]Frameshifting efficiency is shown as mean values and SD from n=3 independent biological replicates (Supplementary Fig. 11c).

[c]Fold change is calculated as the ratio of +1 frameshifting efficiency on $m^1\Psi$-modified to that on unmodified mRNA reporters and is shown as mean values and SD from n=3 independent biological replicates (Supplementary Fig. 11c).

Supplementary References

1 Huter, P. *et al.* Structural Basis for Polyproline-Mediated Ribosome Stalling and Rescue by the Translation Elongation Factor EF-P. *Mol Cell* **68**, 515-527 e516, doi:10.1016/j.molcel.2017.10.014 (2017).

2 Poulis, P., Patel, A., Rodnina, M. V. & Adio, S. Altered tRNA dynamics during translocation on slippery mRNA as determinant of spontaneous ribosome frameshifting. *Nat Commun* **13**, 4231, doi:10.1038/s41467-022-31852-w (2022).
