# Supplementary material for: Recoding by N1-methylpseudouridine guides rational mRNA vaccine design
Source: Nat Commun. 2026 Sep 28;17:10029. doi: 10.1038/s41467-026-77796-3 (PMC13620113; doi:10.1038/s41467-026-77796-3)
Supplement: Supplementary file 3 [file 41467_2026_77796_MOESM3_ESM.pdf]

nature portfolio

Peer Review File

Recoding by N1-methylpseudouridine guides rational mRNA vaccine design

Corresponding Author: Dr Marina Rodnina

This file contains all reviewer reports in order by version, followed by all author rebuttals in order by version.

Version 0:

Reviewer comments:

Reviewer #1

(Remarks to the Author)
This work by Poulis, Robecchi et al. presents smFRET data collected using an in vitro translation system to reveal, at the molecular level, how m1Ψ promotes +1 frameshifting by the ribosome. These experiments are particularly significant because all the U residues are replaced with m1Ψ in current mRNA vaccines, and there is evidence for +1 frameshift products in individuals who have received the COVID-19 vaccine (Mulroney, Willis, Nature 2023). Understanding the molecular drivers of ribosomal frameshifting on m1Ψ-containing mRNAs is essential for the future design of mRNA vaccines and therapeutics.

This elegant study demonstrates that +1 frameshifting is driven by m1Ψ-dependent P-site destabilization. Furthermore, it underscores the importance of mRNA sequence context in frameshifting events. The work is rigorously conducted and well presented. In my opinion, this manuscript is appropriate for Nature Communications, and will be well received and impactful to a broad

Minor comment:

I am particularly interested in the data supporting the idea that "m1Ψm1Ψm1Ψ C drives frameshifting more readily when tRNA supply is limiting." This is an important consideration for the mRNA therapy and vaccine development fields. It may be beneficial to expand on this point by adding a sentence or two discussing its broader implications—for example, how tRNA availability could influence frameshifting in therapeutic settings or vaccine recipients.

Reviewer #2

(Remarks to the Author)
This manuscript investigates the impact of N^1-methylpseudouridine ($m^1\Psi$), a widely used modification in therapeutic and vaccine mRNAs, on translation fidelity, with a particular focus on +1 ribosomal frameshifting. The topic is in principle relevant, given the extensive clinical use of modified mRNAs and the increasing interest in unintended translational recoding events that could generate aberrant peptides with potential immunological consequences. However, in its current form, many conclusions appear overstated, the reporter design is insufficiently justified, and key controls are missing. In addition, there is a lack of discussion placing the results in the context of several previous studies that have already addressed closely related questions using complementary approaches (NAR 2025, PMID: 41099713; Nature Communications, PMID: 39284850; Cell Reports 2022, PMID: 35988540; Nature 2026, PMID: 41535458). These points substantially weaken the interpretation of the data.

Major comments:
P3-L36: It is unclear whether UUU C or UUU U/C motifs are present elsewhere in the mCherry or sfGFP coding sequences. Such motifs could promote ribosomal slippage independently of the intended cassette, thereby contributing to off-target signals and/or GFP production.

P5-L92 (figure 1). The off-target control raises serious concerns regarding data interpretation. In unmodified mRNA, off-target

frameshifting is reported at ~1%, which is unexpectedly high. Conversely, in the m¹Ψ-modified condition, the off-target signal is reduced by approximately 2–3 fold. If the off-target signal is taken as the baseline, most unmodified cassette-containing constructs exhibit frameshifting levels comparable to this baseline, suggesting that in the absence of m¹Ψ, the cassette itself does not induce measurable frameshifting. However, if the reduced off-target baseline in the m¹Ψ condition is considered, then nearly all m¹Ψ-containing constructs appear elevated relative to baseline, which would dramatically change the interpretation of Figure 1e. In this context, the claim of strong context dependency becomes questionable, as the apparent differences may largely reflect changes in the off-target background rather than cassette-specific effects. This issue needs to be addressed explicitly, as it directly affects the core conclusions of the manuscript.

P5-L93: A major conceptual limitation of the study is the absence of direct evidence that GFP fluorescence originates from bona fide +1 frameshifting events. In the current reporter design, it cannot be excluded that GFP expression arises from alternative initiation events upstream of the stop codon cassette, leading to independent translation of the +1-frame ORF. Importantly, no experiment demonstrates that GFP translation is mechanistically coupled to mCherry translation. For instance, introducing a strong stem-loop or other inhibitory element upstream of the mCherry start codon should suppress mCherry translation; under a true frameshifting model, GFP expression should be concomitantly abolished. If GFP fluorescence persists under such conditions, this would strongly argue for independent translation of the two coding sequences rather than frameshifting. Without such controls, the attribution of GFP signal specifically to ribosomal frameshifting remains insufficiently supported.

P6-L105 (ext data Fig 3a). The reported readthrough level of ~1% across two consecutive UGA stop codons is unexpectedly high when compared to the vast literature on stop codon readthrough in human cells, where basal levels are typically around ~0.01%.Using two consecutive UGA codons does not rule out readthrough at one of the two stops, and therefore does not provide a stringent measure of genuine readthrough suppression. This raises concerns about the sensitivity and specificity of the assay, as well as about potential confounding effects intrinsic to the reporter design.

P18-L359. The manuscript claims context-dependent modulation of frameshifting; however, the data do not convincingly support this conclusion. From a mechanistic standpoint, +1 frameshifting minimally requires a four-nucleotide slippery motif. By definition, any functional +1 frameshifting site must therefore contain at least such a motif (e.g., UUU C). If context truly played a role, nucleotides at positions +2 and +3 downstream of the slippery site would be expected to influence frameshifting efficiency. However, the data show no such effect. The absence of any contribution from these positions strongly argues against genuine context dependency and instead suggests that frameshifting is dictated solely by the presence of the minimal four-nucleotide slippery sequence. Consequently, the claim that context modulates frameshifting efficiency appears overstated.

Minor comments
P9 L165. For clarity and ease of reading, it would be helpful if the panels in Extended Data Figure 4 were presented in the same order as they are described in the text.

P19 (figure 4). In Figure 4h, the reported differences are very small, and many biological replicates clearly overlap between conditions. While some differences reach statistical significance, the effect sizes appear minimal, and their biological relevance is uncertain. This should be more clearly acknowledged in the text.

Reviewer #3

(Remarks to the Author)
Summary:

In this exciting and well-written study, the authors investigate the mechanism of m1-pseudouridine-mediated +1 ribosomal frameshifting. Using a combination of in vivo fluorescence reporter assays and single-molecule FRET, they find that m1-pseudouridine-mediated +1 frameshifting occurs when the peptidyl-tRNA pauses in the P-site waiting for the delivery of the next aminoacyl-tRNA. They find that m1-pseudouridine is destabilizing the P-site codon-anticodon interactions thereby promoting +1 frameshifting. In contrast, they find no effect of the modification on decoding or translocation. By replacing the slippery sequences by appropriate alternative codons, frameshifting could be eliminated, thus, providing a rational approach to mitigate the risk of frameshifting in therapeutic mRNAs by codon optimization.
The study is well executed with all the relevant controls performed. After addressing the few comments, this study will be a nice and important publication linking mechanistic biology to translational applications.

Comments (major):

-the authors should discuss in more detail the previous literature on m1-pseudoU frameshifting and elaborate in more detail of what specifically their study adds to this knowledge. For example, what are the specific differences to following previously published paper: https://www.nature.com/articles/s41467-024-51301-0 and likely others? I think the study provides significant advances, they should just be spelled out for a non-expert in this emerging field.

-Fig. 1e: why does off-target have lower frameshifting when m1-pseudoU-modified? This should be discussed.

-number of molecules analyzed should be indicated in all figures (e.g. Fig. 3e)

-methods lines 505-507: for increasing reproducibility, the authors should specify for each reagent in which publication the reagent preparation was described in detail rather than indicating 4 publications at the end of the sentence.

-methods lines 545-547: “A semi-automated algorithm (MATLAB) was used to select single fluorophores showing anticorrelated fluorescence intensities and single-step photobleaching.“ -> the authors should specify in more detail how they did this. This is important as it will affect the FRET efficiency values, which should be comparable to other studies having assigned different ribosome states to specific FRET efficiencies.

-methods 555-552: the authors should explain the metric they used to determine the number of states (number of gaussians they used to fit the data)

Comments (minor):

-147: sentence is missing verb
-191-194: sentence should be rewritten for correct grammar and clarity

Version 1:

Reviewer comments:

Reviewer #2

(Remarks to the Author)
The authors have addressed most of my first-round concerns satisfactorily, including the internal-initiation controls (mutation of the +1-frame AUG and the T2A near-cognate CUG), for which I am satisfied. The remaining points below concern the calibration of the conclusions and the completeness of reporting rather than the validity of the experiments, and none of them requires substantial new experimentation.

I have two important comments for the authors:
1- The entire biophysical and kinetic core of the paper, the smFRET, the reconstituted translation, the drop-off kinetics, and the tRNA-limitation titrations, is performed in an E. coli system, whereas the phenomenon and its clinical relevance are eukaryotic. The justification given (lines 146-149) that decoding is conserved is valid for the codon-recognition step but does not automatically extend to reading-frame maintenance or to the quantitative propensity for +1 slippage, which are not established to be equivalent between bacterial and mammalian ribosomes. This matters because the cellular data do not confirm the proposed mechanism: Fig. 4g reports no correlation between the m1Ψ-dependent frameshifting response and the ribosome A-site dwell time in HEK293T cells, and in their reply to Reviewer 1 the authors state they found no correlation with cognate tRNA availability in cells. A tRNA-limited P-site stalling mechanism therefore has clear in vitro support but no positive cellular validation. The manuscript should state explicitly that the mechanism is demonstrated in the bacterial system and inferred, not shown, for human translation, and should reconcile the strong in vitro tRNA dependence with the absent cellular correlation rather than presenting both without comment.

2- "Context" framing. My point on positions +2 and +3 has been partly conceded, but the framing remains loose. The nucleotide immediately 3' of UUU is part of the slippery-motif definition itself (UUUC versus UUUU), not a context surrounding a fixed motif (the minimal length for a slippery sequence is 4nt), and the authors confirm no contribution from positions +2 or +3. The C-versus-U effect in Fig. 4h is real and statistically significant, but it should be described as the identity of the fourth motif nucleotide rather than as general "codon context" modulation (lines 336, 365, 421-423). Please confirm that the abstract and Discussion wording were revised to match the narrower claim stated in the point-by-point reply.

Reviewer #3

(Remarks to the Author)
The authors have addressed all my requests.

Open Access This Peer Review File is licensed under a Creative Commons Attribution 4.0 International License, which permits use, sharing, adaptation, distribution and reproduction in any medium or format, as long as you give appropriate credit to the original author(s) and the source, provide a link to the Creative Commons license, and indicate if changes were made.
In cases where reviewers are anonymous, credit should be given to 'Anonymous Referee' and the source.
The images or other third party material in this Peer Review File are included in the article's Creative Commons license, unless indicated otherwise in a credit line to the material. If material is not included in the article's Creative Commons license and your intended use is not permitted by statutory regulation or exceeds the permitted use, you will need to obtain permission directly from the copyright holder.
To view a copy of this license, visit https://creativecommons.org/licenses/by/4.0/

Point-by-point reply to Reviewer Comments for NCOMMS-26-000168-T-R1

REVIEWER COMMENTS

Reviewer #1 (Remarks to the Author):

This work by Poulis, Robecchi et al. presents smFRET data collected using an in vitro translation system to reveal, at the molecular level, how m1Ψ promotes +1 frameshifting by the ribosome. These experiments are particularly significant because all the U residues are replaced with m1Ψ in current mRNA vaccines, and there is evidence for +1 frameshift products in individuals who have received the COVID-19 vaccine (Mulroney, Willis, Nature 2023). Understanding the molecular drivers of ribosomal frameshifting on m1Ψ-containing mRNAs is essential for the future design of mRNA vaccines and therapeutics.

This elegant study demonstrates that +1 frameshifting is driven by m1Ψ-dependent P-site destabilization. Furthermore, it underscores the importance of mRNA sequence context in frameshifting events. The work is rigorously conducted and well presented. In my opinion, this manuscript is appropriate for Nature Communications, and will be well received and impactful to a broad

Reply: We thank the reviewer for the positive comments on the manuscript.

Minor comment:

I am particularly interested in the data supporting the idea that "m1Ψm1Ψm1Ψ C drives frameshifting more readily when tRNA supply is limiting." This is an important consideration for the mRNA therapy and vaccine development fields. It may be beneficial to expand on this point by adding a sentence or two discussing its broader implications—for example, how tRNA availability could influence frameshifting in therapeutic settings or vaccine recipients.

Reply: We thank the reviewer for this important remark. Our *in-vitro* translation experiments indeed suggest that frameshifting is promoted under conditions of limiting tRNA supply and may therefore be modulated by cellular tRNA abundance. However, in our HEK293T cell reporter assay we did not find a correlation between +1 frameshifting efficiency and the availability of the cognate tRNA (Fig. 4g). Nevertheless, we agree that the broader potential implications for mRNA therapeutics and vaccine development are important. To address this point, we have expanded the Discussion and added a statement highlighting the possible impact of cellular tRNA availability on frameshifting in therapeutic context (lines 423-434).

Reviewer #2 (Remarks to the Author):

This manuscript investigates the impact of N^1-methylpseudouridine (m^{1}Ψ), a widely used modification in therapeutic and vaccine mRNAs, on translation fidelity, with a particular focus on +1 ribosomal frameshifting. The topic is in principle relevant, given the extensive clinical use of modified mRNAs and the increasing interest in unintended translational recoding events that could generate aberrant peptides with potential immunological consequences. However, in its current form, many conclusions appear overstated, the reporter design is insufficiently justified, and key controls are missing. In addition, there is a lack of discussion placing the results in the context of several previous studies that have already addressed closely related questions using complementary approaches (NAR 2025, PMID: 41099713; Nature Communications, PMID: 39284850; Cell Reports 2022, PMID: 35988540; Nature 2026, PMID: 41535458). These points substantially weaken the interpretation of the data.

Reply: We thank the reviewer for highlighting these studies and the importance of placing our findings in the context of previous work.

We respectfully note that the *Nucleic Acid Research* study (2025, PMID: 41099713), is not directly relevant to our work, as it focuses on the sensing of double-stranded RNA IVT byproducts and their mitigation by *N^1*-methylpseudouridine on early zebrafish embryos, rather than on translation fidelity or recoding events.

We also respectfully note that the study in *Cell Reports* (2022, PMID: 35988540) primarily addresses miscoding during translation of m^{1}Ψ-modified mRNAs. In that work, translation products were isolated via a C-terminal FLAG tag located in the 0-reading frame. Because frameshifted translation products would terminate at out-of-frame stop codons upstream of the FLAG tag, such products would not be recovered for mass spectrometric analysis. We have nevertheless added a statement discussing this study and its relation to more recent studies (lines 50-51).

We agree that the study in *Nature Communications* (PMID: 39284850), reporting modest to no effect of m^{1}Ψ on cognate tRNA decoding, are consistent with our results on decoding dynamics on unmodified and m^{1}Ψ-modified codons (Supplementary Fig. 4 and 5). We have added a statement noting this agreement (lines 184-185).

Finally, we agree that the *Nature* study (2026, PMID: 41535458) is highly relevant to our work. As it was published after the initial submission of our manuscript, we could not cite it before. We have now incorporated this study into the revised manuscript and added statements highlighting the agreement between the two studies regarding both the extent

of frameshifting (lines 105-107) and the effects of m¹Ψ in codon-anticodon interactions (lines 438-441).

Major comments:

P3-L36: It is unclear whether UUU C or UUU U/C motifs are present elsewhere in the mCherry or sfGFP coding sequences. Such motifs could promote ribosomal slippage independently of the intended cassette, thereby contributing to off-target signals and/or GFP production.

Reply: We thank the reviewer for raising this point. Indeed, the *mChy* ORF contains UUUC motifs at Phe codons 92 and 134, and the *sfGFP* ORF contains UUUU motifs at Phe codons 27, 71 and 83. No UUUU/C motifs are present within the P2A or T2A StopGo elements. We agree that such slippery sequences could, in principle, contribute to frameshifting. However, +1 frameshifting within the *mCherry* ORF would encounter a +1-frame stop codon within the downstream P2A element, preventing translation from continuing towards *sfGFP*. In addition, to account for potential +1 frameshifting arising from the T2A or endogenous slippery sequences within the *sfGFP* ORF, we included a control reporter lacking the test cassette (Fig. 1e & Supplementary Table 1). This background signal was used to define the lower detection limit of the assay and to distinguish cassette-dependent frameshifting from reporter-intrinsic events.

P5-L92 (figure 1). The off-target control raises serious concerns regarding data interpretation. In unmodified mRNA, off-target frameshifting is reported at ~1%, which is unexpectedly high. Conversely, in the m¹Ψ-modified condition, the off-target signal is reduced by approximately 2–3 fold. If the off-target signal is taken as the baseline, most unmodified cassette-containing constructs exhibit frameshifting levels comparable to this baseline, suggesting that in the absence of m¹Ψ, the cassette itself does not induce measurable frameshifting. However, if the reduced off-target baseline in the m¹Ψ condition is considered, then nearly all m¹Ψ-containing constructs appear elevated relative to baseline, which would dramatically change the interpretation of Figure 1e. In this context, the claim of strong context dependency becomes questionable, as the apparent differences may largely reflect changes in the off-target background rather than cassette-specific effects. This issue needs to be addressed explicitly, as it directly affects the core conclusions of the manuscript.

Reply: We agree with the reviewer that the background signal differs between unmodified and m¹Ψ-modified reporter mRNAs (Fig. 1e). At present, we do not a mechanistic explanation for this difference. To address this issue explicitly, we now define the signal measured with the control reporter lacking the test cassette as the background signal of the assay (lines 82-83; Fig. 1e & Supplementary Table 1).

One possible approach would be to subtract the background signal from the values obtained with the test constructs. However, we chose not to do so because the origin of the background signal is currently unknown. The observation that the background signal differs between unmodified and $m^1\Psi$-modified reporter mRNAs suggests that it may reflect a biologically meaningful effect of $m^1\Psi$ on reporter expression rather than a purely technical artifact. We therefore felt that subtracting or otherwise correcting for this signal could obscure potentially important information and would introduce assumptions that are not presently supported by experimental evidence. Instead, we report the raw measurements and explicitly disclose the background signal to allow readers to assess its impact on the data interpretation.

We respectfully disagree, however, that the difference in background signal alters the central conclusions of the manuscript. While normalization to the lower background signal observed for $m^1\Psi$-modified reporters would increase the apparent magnitude of frameshifting for all $m^1\Psi$-containing constructs, it would not affect the relative differences among slippery sequences shown in Fig. 1e. Likewise, the nucleotide-specific effects of $m^1\Psi$ on frameshifting, including the distinct behavior of codons containing a first-position C versus U (Fig. 4h), remain unchanged. Therefore, although the origin of the differing background signals remains unresolved, the sequence-specific and nucleotide-specific trends that form the basis of our conclusions are unaffected.

P5-L93: A major conceptual limitation of the study is the absence of direct evidence that GFP fluorescence originates from bona fide +1 frameshifting events. In the current reporter design, it cannot be excluded that GFP expression arises from alternative initiation events upstream of the stop codon cassette, leading to independent translation of the +1-frame ORF. Importantly, no experiment demonstrates that GFP translation is mechanistically coupled to mCherry translation. For instance, introducing a strong stem-loop or other inhibitory element upstream of the mCherry start codon should suppress mCherry translation; under a true frameshifting model, GFP expression should be concomitantly abolished. If GFP fluorescence persists under such conditions, this would strongly argue for independent translation of the two coding sequences rather than frameshifting. Without such controls, the attribution of GFP signal specifically to ribosomal frameshifting remains insufficiently supported.

Reply: We thank the reviewer for this important comment and for suggesting additional controls to exclude alternative mechanisms of sfGFP expression. To directly address the possibility of internal initiation, we systematically removed potential initiation sites within the reporter construct while maintaining normal translation of the upstream mCherry ORF. Specifically, we mutated the AUG start codon of the +1-frame sfGFP ORF and the near-cognate CUG codon present in the T2A element to the synonymous non-cognate codon CUU (Supplementary Fig. 1e). These mutations did not affect the observed +1 frameshifting signal

compared to the corresponding reporter constructs (Fig. 1e and Supplementary Table 1). These results exclude initiation from the identified cognate and near-cognate start codons as the source of sfGFP expression.

In addition, alternative initiation events upstream of the test cassette cannot account for sfGFP production because translation initiated in the +1 frame upstream of the cassette would encounter a stop codon in the +1 frame within the P2A element and terminate before reaching sfGFP.

We also considered the reviewer's suggestion to inhibit mCherry translation by introducing a strong secondary structure upstream of the mCherry start codon. However, such a construct would not specifically distinguish frameshifting from internal initiation. In cap-dependent mRNAs, stable secondary structures near the 5′ end impair ribosomal scanning and reduce access not only to the mCherry start codon but also to any downstream initiation sites. Consequently, loss of both mCherry and sfGFP expression would be expected regardless of whether sfGFP originates from frameshifting or internal initiation, making this experiment difficult to interpret.

Finally, as an independent line of evidence, we note that sfGFP expression is tightly correlated with mCherry expression when the two ORFs are placed in frame (Supplementary Fig. 1c,d, orange dot plots), whereas this correlation is lost when sfGFP is placed out of frame (Supplementary Fig. 1c,d, blue dot plots). Together with the internal-initiation controls described above, these results support the conclusion that sfGFP production in our reporter system reflects translation coupled to the upstream ORF and is not caused by unintended internal initiation. We have now included the additional initiation-control experiments in Supplementary Fig. 1e and described them in the revised manuscript (lines 92–95).

P6-L105 (ext data Fig 3a). The reported readthrough level of ~1% across two consecutive UGA stop codons is unexpectedly high when compared to the vast literature on stop codon readthrough in human cells, where basal levels are typically around ~0.01%.Using two consecutive UGA codons does not rule out readthrough at one of the two stops, and therefore does not provide a stringent measure of genuine readthrough suppression. This raises concerns about the sensitivity and specificity of the assay, as well as about potential confounding effects intrinsic to the reporter design.

Reply: We thank the reviewer for the comment. We agree that the apparent readthrough values observed in our reporter system are higher than the lowest basal stop-codon readthrough efficiencies reported in the literature. However, published measurements of stop-codon readthrough in human cells span a broad range, from approximately 0.01–0.5% (https://doi.org/10.1371/journal.pgen.1002608) to 0.1-2.91% (https://doi.org/10.7554/eLife.03640), depending on the stop-codon context and experimental system. Thus, the value of 0.01% cited by the reviewer represents the lower end of the reported range rather than a generally observed basal readthrough level.

Importantly, we did not intend these experiments to provide a stringent quantitative measure of stop-codon readthrough efficiency or to demonstrate complete suppression of readthrough by two consecutive UGA codons. Rather, our goal was to determine whether m¹Ψ promotes readthrough in the sequence context of the original FDA-approved BNT162b2 mRNA, which contains two consecutive UGA stop codons. Within the sensitivity limits of our assay, we did not detect an increase in signal above background for either unmodified or m¹Ψ-modified reporters (Supplementary Fig. 3 and Supplementary Table 1). We therefore conclude that m¹Ψ does not promote readthrough of the BNT162b2 stop codon region to levels detectable in our system, i.e. above the approximately 1% background signal of the assay. To avoid overinterpretation, we have clarified this point in the revised manuscript (lines 111–114).

P18-L359. The manuscript claims context-dependent modulation of frameshifting; however, the data do not convincingly support this conclusion. From a mechanistic standpoint, +1 frameshifting minimally requires a four-nucleotide slippery motif. By definition, any functional +1 frameshifting site must therefore contain at least such a motif (e.g., UUU C). If context truly played a role, nucleotides at positions +2 and +3 downstream of the slippery site would be expected to influence frameshifting efficiency. However, the data show no such effect. The absence of any contribution from these positions strongly argues against genuine context dependency and instead suggests that frameshifting is dictated solely by the presence of the minimal four-nucleotide slippery sequence. Consequently, the claim that context modulates frameshifting efficiency appears overstated.

Reply: We thank the reviewer for this comment. We believe that the apparent discrepancy arises from a different interpretation of the term "context". In our study, the context of the slippery UUU sequence includes the nucleotide immediately following it, i.e., the first nucleotide of the downstream codon. Using this definition, our data demonstrate a clear context effect. Specifically, m¹Ψ increases frameshifting on UUUC motifs but not on UUUU motifs (Fig. 4h), indicating that the identity of the nucleotide immediately following the slippery sequence influences frameshifting efficiency. This observation is consistent with previous work showing that $tRNA^{Phe}$ has a thermodynamic preference for pairing with UUC rather than UUU codons (Bock et al., 2019, DOI: 10.1038/s41467-019-12648-x).

We agree that we do not observe additional contributions from nucleotides located further downstream. However, this does not negate the context dependence observed at the nucleotide immediately adjacent to the slippery sequence. To avoid ambiguity, we have revised the manuscript to clarify that the observed context dependence refers specifically to the nucleotide at the +1 position relative to the slippery UUU sequence (lines 372-373).

Minor comments

P9 L165. For clarity and ease of reading, it would be helpful if the panels in Extended Data Figure 4 were presented in the same order as they are described in the text.

Reply: We thank the reviewer for this suggestion. The panels in Supplementary Fig. 4 are presented in the same order as they are described in the text. However, we recognize that the arrangement of panels 4e-j, which are displayed vertically rather than horizontally, may make the organization less immediately apparent. We chose this layout to facilitate direct comparison of the timing of key decoding events between unmodified and $m^1\Psi$-modified codons.

P19 (figure 4). In Figure 4h, the reported differences are very small, and many biological replicates clearly overlap between conditions. While some differences reach statistical significance, the effect sizes appear minimal, and their biological relevance is uncertain. This should be more clearly acknowledged in the text.

Reply: We would like to clarify that the individual data points shown in Fig. 4h do not represent biological replicates. Rather, each point corresponds to the mean fold change in +1 frameshifting measured for a distinct codon, calculated from three independent biological replicates. The purpose of this analysis was to compare codons grouped according to the identity of the first nucleotide downstream of the slippery sequence.

We agree that the observed differences are modest in magnitude. Accordingly, we do not interpret Fig. 4h as evidence for a large effect of sequence context on frameshifting. Instead, the analysis reveals a consistent nucleotide-dependent trend, namely that codons with a first-position C show a greater $m^1\Psi$-dependent increase in frameshifting than codons with a first-position U, which is a statistically significant trend.

Reviewer #3 (Remarks to the Author):

Summary:

In this exciting and well-written study, the authors investigate the mechanism of m1-pseudouridine-mediated +1 ribosomal frameshifting. Using a combination of in vivo fluorescence reporter assays and single-molecule FRET, they find that m1-pseudouridine-mediated +1 frameshifting occurs when the peptidyl-tRNA pauses in the P-site waiting for the delivery of the next aminoacyl-tRNA. They find that m1-pseudouridine is destabilizing the P-site codon-anticodon interactions thereby promoting +1 frameshifting. In contrast, they find no effect of the modification on decoding or translocation. By replacing the slippery sequences by appropriate alternative codons, frameshifting could be eliminated, thus, providing a rational approach to mitigate the risk of frameshifting in therapeutic mRNAs by codon optimization.

The study is well executed with all the relevant controls performed. After addressing the few comments, this study will be a nice and important publication linking mechanistic biology to translational applications.

Reply: We thank the reviewer for the positive comments on the manuscript.

Comments (major):

-the authors should discuss in more detail the previous literature on m1-pseudoU frameshifting and elaborate in more detail of what specifically their study adds to this knowledge. For example, what are the specific differences to following previously published paper: https://www.nature.com/articles/s41467-024-51301-0 and likely others? I think the study provides significant advances, they should just be spelled out for a non-expert in this emerging field.

Reply: We thank the reviewer for this constructive suggestion and for emphasizing the importance of clearly positioning our work within the rapidly emerging literature on $m^1\Psi$-induced translation errors. We have expanded the Introduction and Discussion to place our findings in the context of four recent studies published between 2024 and 2026 that reported translation errors associated with $m^1\Psi$-modified mRNAs by adding the 2026 Rozman paper. Specifically, we now discuss the studies by Mulroney et al. (2024; PMID: 38057663), Stiving et al. (2025; PMID: 39988579), Monroe et al. (2024; PMID: 39284850), and Rozman et al. (2026; PMID: 41535458), highlighting both areas of agreement and the distinct contributions of our work.

Our results agree with Mulroney et al. (2024) and Stiving et al. (2025) in demonstrating that $m^1\Psi$ can promote +1 frameshifting. We now explicitly discuss these studies and highlight that, while the qualitative conclusions are consistent, the magnitude of frameshifting differs from that reported by Mulroney et al. (2024). We attribute this difference to the distinct reporter constructs and sequence contexts used in the two studies (lines 103–105).

Our results also agree with Monroe et al. (2024), who reported little or no effect of $m^1\Psi$ on cognate tRNA decoding. Consistent with their conclusions, our single-molecule FRET measurements of cognate tRNA decoding kinetics revealed no substantial differences between unmodified and $m^1\Psi$-modified codons (Supplementary Figs. 4 and 5; lines 184–185).

Finally, we discuss the recent study by Rozman et al. (2026), which reports comparable levels of $m^1\Psi$-induced frameshifting. We further relate their structural observations to our mechanistic findings by highlighting how $m^1\Psi$-dependent perturbations of codon–anticodon interactions may promote frameshifting (lines 438–441).

Importantly, beyond confirming and extending observations from these recent studies, our work provides several advances. First, we identify sequence determinants that modulate the frameshifting response to m¹Ψ. Second, by combining cellular reporter assays with fully reconstituted in vitro translation systems, we show that m¹Ψ-induced frameshifting is linked to destabilization of codon–anticodon interactions and is enhanced under conditions of limiting tRNA availability. Third, our kinetic analyses distinguish effects on translation fidelity from effects on cognate tRNA decoding, thereby providing a mechanistic framework for understanding how m¹Ψ promotes recoding.

We thank the reviewer for their positive comments regarding the significance of our study. We have expanded the Discussion to more clearly highlight the broader implications of our findings. Specifically, we discuss the potential role of codon optimization not only in improving expression and translation fidelity of therapeutic mRNAs, but also in modulating translation outcomes in cellular contexts with altered tRNA pools, such as cancer (lines 423-434). In addition, we emphasize that translation fidelity should be considered alongside immunogenicity and expression efficiency when evaluating the suitability of mRNA modifications for therapeutic and vaccine applications (463-466).

-Fig. 1e: why does off-target have lower frameshifting when m1-pseudoU-modified? This should be discussed.

Reply: We thank the reviewer for raising this point. We agree that the lower background signal observed with m¹Ψ-modified reporter mRNAs compared to unmodified reporters is an interesting observation. At present, we do not have direct evidence that would explain the origin of this difference, and we therefore refrain from speculating on the underlying mechanism.

To address this issue explicitly, we now discuss this observation in the manuscript and define the signal measured with the control reporter lacking the test cassette (1.03 ± 0.05%; Fig. 1e and Supplementary Table 1) as the background signal of the assay. Because the origin of this signal is unknown, we chose not to subtract it from the measurements obtained with the test constructs. Instead, we report the raw data and make the background signal explicit, allowing readers to assess its impact on the interpretation of the results.

Importantly, although the background signal differs between unmodified and m¹Ψ-modified reporter mRNAs, the sequence-dependent trends and nucleotide-specific effects reported in our study remain unchanged.

-number of molecules analyzed should be indicated in all figures (e.g. Fig. 3e)

Reply: We have revised the legends of Fig. 3e and Supplementary Fig. 5a,d to include the number of molecules analyzed in each experiment.

-methods lines 505-507: for increasing reproducibility, the authors should specify for each reagent in which publication the reagent preparation was described in detail rather than indicating 4 publications at the end of the sentence.

Reply: We have revised the Methods section to pair each reagent with the specific reference describing its preparation.

-methods lines 545-547: “A semi-automated algorithm (MATLAB) was used to select single fluorophores showing anticorrelated fluorescence intensities and single-step photobleaching.“ -> the authors should specify in more detail how they did this. This is important as it will affect the FRET efficiency values, which should be comparable to other studies having assigned different ribosome states to specific FRET efficiencies.

Reply: We thank the reviewer for this comment. We agree that the criteria used for trajectory selection are important for the interpretation and comparison of FRET states. We have therefore expanded the Methods section to describe the analysis workflow in greater detail (lines 577-581).

-methods 555-552: the authors should explain the metric they used to determine the number of states (number of gaussians they used to fit the data)

Reply: We thank the reviewer for this comment. We have expanded the Methods section to clarify how the number of Gaussian components was determined. Briefly, the number of states was established by progressively increasing the number of Gaussian components and evaluating the resulting fits. We selected the minimal number of components that adequately described the FRET distributions, minimized residuals, and yielded reproducible state parameters (mean FRET values and variances) across independent datasets. Additional components did not substantially improve the fit quality and typically resulted in unstable or poorly resolved parameters, indicating overfitting.

As an independent validation, Hidden Markov modeling of individual trajectories identified the same number of dominant FRET states. Furthermore, dwell-time analyses of the corresponding transitions were consistent with these state assignments. We have now included these criteria in the revised Methods section (lines 584-590).

Comments (minor):

-147: sentence is missing verb

Reply: We have modified the sentence to include a verb.

-191-194: sentence should be rewritten for correct grammar and clarity

Reply: We have modified the sentence for clarity.

Point-by-point reply to Reviewer Comments for NCOMMS-26-000168A

REVIEWER COMMENTS

Reviewer #2 (Remarks to the Author):

The authors have addressed most of my first-round concerns satisfactorily, including the internal-initiation controls (mutation of the +1-frame AUG and the T2A near-cognate CUG), for which I am satisfied. The remaining points below concern the calibration of the conclusions and the completeness of reporting rather than the validity of the experiments, and none of them requires substantial new experimentation.

Reply: We thank the reviewer for the positive comments.

I have two important comments for the authors:

1- The entire biophysical and kinetic core of the paper, the smFRET, the reconstituted translation, the drop-off kinetics, and the tRNA-limitation titrations, is performed in an E. coli system, whereas the phenomenon and its clinical relevance are eukaryotic. The justification given (lines 146-149) that decoding is conserved is valid for the codon-recognition step but does not automatically extend to reading-frame maintenance or to the quantitative propensity for +1 slippage, which are not established to be equivalent between bacterial and mammalian ribosomes. This matters because the cellular data do not confirm the proposed mechanism: Fig. 4g reports no correlation between the m1Ψ-dependent frameshifting response and the ribosome A-site dwell time in HEK293T cells, and in their reply to Reviewer 1 the authors state they found no correlation with cognate tRNA availability in cells. A tRNA-limited P-site stalling mechanism therefore has clear in vitro support but no positive cellular validation. The manuscript should state explicitly that the mechanism is demonstrated in the bacterial system and inferred, not shown, for human translation, and should reconcile the strong in vitro tRNA dependence with the absent cellular correlation rather than presenting both without comment.

Reply: We agree with the reviewer that the tRNA-limitation effect can only be inferred, and is not yet shown, in human translation. For that reason, we have added a statement in the Discussion that the effect can be inferred for human translation (lines 298-299).

2- "Context" framing. My point on positions +2 and +3 has been partly conceded, but the framing remains loose. The nucleotide immediately 3' of UUU is part of the slippery-motif definition itself (UUUC versus UUUU), not a context surrounding a fixed motif (the minimal length for a slippery sequence is 4nt), and the authors confirm no contribution

from positions +2 or +3. The C-versus-U effect in Fig. 4h is real and statistically significant, but it should be described as the identity of the fourth motif nucleotide rather than as general "codon context" modulation (lines 336, 365, 421-423). Please confirm that the abstract and Discussion wording were revised to match the narrower claim stated in the point-by-point reply.

Reply: We have now revised and explicitly stated, in the Results and Discussion, that the effect depends on the fourth nucleotide of the slippery sequence and refrained from using the general term ‘codon context’ (line 58, 87, 276 and 325).

Reviewer #3 (Remarks to the Author):

The authors have addressed all my requests.

Reply: We thank the reviewer for their critical comments that substantially improved the quality of the manuscript.
